# End Users’ and Other Stakeholders’ Needs and Requirements in the Development of a Personalized Integrated Care Platform (PROCare4Life) for Older People With Dementia or Parkinson Disease: Mixed Methods Study

**DOI:** 10.2196/39199

**Published:** 2022-11-30

**Authors:** Mona Ahmed, Mayca Marín, Daniella How, Elda Judica, Peppino Tropea, Ellen Bentlage, Joaquim J Ferreira, Raquel Bouça-Machado, Michael Brach

**Affiliations:** 1 Institute of Sport and Exercise Sciences Münster University Münster Germany; 2 Asociación Parkinson Madrid (APM) Madrid Spain; 3 Department of Neurorehabilitation Sciences Casa di Cura del Policlinico Milan Italy; 4 Campus Neurológico Sénior (CNS) Torres Vedras Portugal

**Keywords:** neurodegenerative, Parkinson disease, dementia, chronic diseases, health care technologies, integrated care, information and communication technology, ICT, user-centered design, mobile phone

## Abstract

**Background:**

With what has been known as the “*triple-win effect*”, introducing information and communication technologies (ICTs) in the health care of neurodegenerative diseases is beneficial in delaying the need for institutional care, reducing the associated health care costs, reducing the caregiving burden, and improving individuals’ quality of life. Nevertheless, the mismatch between the users’ expectations and their actual needs remains one of the main challenges that can reduce the usability of technology solutions. Therefore, the European project Personalized Integrated Care Promoting Quality of Life for Older People (PROCare4Life), which aimed to develop an ICT-based platform for all parties involved in the health care of neurodegenerative diseases, adopted a user-centered design approach, where all users are involved from the inception and throughout the platform development and implementation to integrate their needs and requirements in the proposed platform.

**Objective:**

This paper presents the results of a study on the needs and requirements of the potential end users (older people with neurodegenerative diseases, caregivers, and health care professionals) and other key stakeholders in the development of the PROCare4Life platform.

**Methods:**

A mixed qualitative and quantitative study design was used, including 2 web-based surveys, 40 interviews, and 4 workshops. The study was conducted between April and September 2020 in 5 European countries: Germany, Italy, Portugal, Romania, and Spain. Both data types were analyzed separately and then merged and interpreted, with greater priority placed on qualitative research.

**Results:**

A total of 217 participants were recruited; 157 (72.4%) of them completed the web-based surveys (n=85, 54.1% patients and n=72, 45.9% caregivers), and 60 (27.6%) individuals participated in the qualitative research (20/60, 33% health care professionals; 5/60, 8% patients; 5/60, 8% caregivers; and 30/60, 50% key stakeholders). We identified 3 main themes (T): (T1) experiences associated with illness, (T2) thoughts about the platform technology, and (T3) desired properties. Alerts for adverse events, communication tools, reminders, and monitoring are constantly needed functionalities, whereas ease of use, personalization, and user-friendliness are foreseen as necessary features.

**Conclusions:**

This paper identified the key personal, social, and health factors that influence the daily lives of the potential end users and reflected on their needs and expectations regarding the design of the proposed PROCare4Life platform. The collected data were useful for the development of the PROCare4Life platform. Although the combination and collection of features for diverse user groups are typical for integrated care platforms, it results in exponential complexity for designers, developers, and users. Contradicting opinions and several concerns in this study demonstrate that an ICT-integrated care platform should not promise too much for too many. Instead, selection, focus, and, sometimes, restriction to essentials are necessary. Users and other stakeholders should be involved in these decisions.

**International Registered Report Identifier (IRRID):**

RR2-10.2196/22463

## Introduction

### Background

Neurodegenerative diseases (NDDs), including dementia and Parkinson disease (PD), are among the most common chronic diseases associated with aging [1]. Characterized by a continuous decline in motor and cognitive abilities [2], difficulties in performing daily activities, and altered behavior [3], NDDs are mostly disabling diseases that negatively impact the quality of life of older populations and their families [1,4]. With the increased prevalence of NDDs, an enormous burden is placed on health care systems in terms of both resources and costs [2,5-9]. Therefore, implementing alternative health care solutions is needed [10].

Integrated care, which coordinates and brings together different health services, has the advantages of optimizing health care resources and being able to respond to the needs of older populations with chronic diseases [11,12]. In Europe, as a part of the eHealth action plan in supporting active aging, the introduction of the integrated care jointly with information and communication technology (ICT) supportive tools has contributed to improving patient experience and providing more efficient health care services at lower costs [13,14]. Divided into wearable, nonwearable, and hybrid-based categories, ICTs offer a wide variety of technological solutions with the purpose of either monitoring or managing the users’ health [15].

In the care of NDDs, with what has been known as the “*triple-win effect,*” ICTs are beneficial in “(1) delaying the need for institutional care, and reduction of the associated health care costs, (2) reducing the caregiving burden and (3) improving individuals’ QoL by helping to keep an independent lifestyle, autonomy and social interaction” [16]. In addition, the integration of health-related data of patients in an interactive web interface enables health care professionals (HCPs) to better monitor and support their patients [17,18].

Although older people, their families, and HCPs are positive about using ICTs [19], the mismatch between the users’ expectations and their actual needs remains one of the main challenges that can reduce the usability of technology solutions targeting older people with mild cognitive decline [20]. In fact, developing an ill-fitting ICT for this target group can be a burden instead of being a supportive tool [21,22]. Therefore, it is crucial to first identify the needs of the users when implementing ICTs in health care to better develop a suitable solution [23].

In this paper, we present the results of a study on the needs and requirements of older people with NDDs, their caregivers, HCPs, and other key stakeholders in the development of a personalized integrated ICT-based, Personalized Integrated Care Promoting Quality of Life for Older People Platform (PROCare4Life). These results ought to drive implications on the design and the properties of the platform.

### PROCare4Life

PROCare4Life is an ICT-based, integrated, scalable, and interactive health care platform. The intended end users include older people with NDDs, caregivers, and HCPs involved in the care process. The PROCare4Life platform plans to collect disease, cognitive, and behavior related data about the patients via wearables, stationary devices, medical records, and other sensors. In a highly secured and protected cloud environment, algorithms analyze and process these data to create a profile for each patient. On the basis of this profile, personalized information and recommendations are provided to those involved in the care plan. The end users will be able to interact with a wide range of services via various digital devices such as smartphone, tablet, or smart television. More details about the aims and the technology of PROCare4Life are reported elsewhere [24].

Throughout the entire development process of the PROCare4Life platform, the project adopted a user-centered design (UCD) approach [25]. This has been recommended for decision makers and leaders in the process of developing ICT in integrated care [26] to ensure active engagement and incorporation of the intended users’ feedback. In line with this approach, the following steps were incorporated: (1) study of the user needs and requirements; (2) iterative design throughout the pilot phases; (3) iterative user evaluation, refining the design throughout the pilots; and (4) a final product that is developed based on the iterative cycles and evaluation or a developed final product based on the iterative cycles and evaluation. This study focused on the first step, understanding the users’ needs and requirements.

### Research on Users’ Needs

In general, ICT solutions need to be easy to use, private, secure, and affordable in terms of costs [27]. However, older people tend to have heterogeneous needs [28], with possible conflicts among patients, caregivers, and HCPs [29]. In dementia, previous studies have summarized the main need areas as information, company, memory and daily activity support, and reduction of psychological stress [30,31]. A systematic review by Lauriks et al [32] that aimed to identify the unmet needs of patients and caregivers stated that ICTs need to be personalized according to the users’ needs and abilities. Boman et al [33] studied the needs of people with cognitive impairment in the design of an ICT-based device. The study reported that the participants were positive about including calendars as memory support, whereas HCPs valued a feature that allows them to view the previous and current care plans. However, low participant numbers were reported as one of the study limitations. In PD, clinical symptoms have high daily fluctuations, meaning that nonmotor- and motor-related symptoms vary within and between days [34]. Therefore, the ICT solution needs to be able to monitor and identify all relevant changes and develop personalized strategies to counteract them [35]. In addition, social support positively affects the ability of patients with PD to cope with the difficulties in daily living and reduces the risk of developing nonmotor symptoms, such as depression [36].

The critique of previous research was that it did not consider the users in the early stages of development. Both patients and caregivers were included at later stages, which resulted in the lost value of their experiences [37].

Following the multidisciplinary principle of the UCD approach, in addition to the intended end users identified as patients, caregivers, and HCPs, the PROCare4Life study on users’ needs includes other key stakeholders from different related health care disciplines. These stakeholders are academic researchers, decision makers, markets, and media actors. The overall objectives of this study were as follows:

1. Collecting detailed information on the opinions, thoughts, experiences, and feelings of the end users (patients, caregivers, and HCPs) and other key stakeholders regarding NDDs, health care processes, and digital health care solutions to identify those aspects where the PROCare4Life platform would best suit and support them.

2. Identifying the aspects that the PROCare4Life platform should consider to achieve success in its acceptance, development, and marketing (eg, strengths and weaknesses, factors that influence the digital health care market, and communication channels through which to adequately diffuse the product).

This paper presents the results related to the first aim of the study on users’ needs and requirements.

## Methods

### Study Design

In a mixed methods study design, we followed the formative and summative research methodologies to identify and analyze the end user needs (identified as patients, caregivers, and HCPs), key stakeholder perspectives (identified as academic researchers, media actors, policy makers, and market actors), and context. In the 2-step approach, we applied both qualitative and quantitative research methods. The quantitative data included 2 web-based surveys involving patients and caregivers. The qualitative data included semistructured interviews and workshops involving the end users and other key stakeholders. This study placed greater priority on qualitative research, with quantitative research playing a supportive role [38].

### Study Procedure and Eligibility Criteria

This study was conducted between April and September 2020 in 5 European countries: Germany, Italy, Portugal, Romania, and Spain. The web-based surveys were launched first, followed by the semistructured interviews. Finally, 4 workshops that involved HCPs were conducted, in which the preliminary results from the surveys were presented and discussed.

In this study, patients were included if they were aged ≥65 years and diagnosed with PD or dementia, including Alzheimer disease and other dementias (OD). Patients with substantial cognitive impairment, intellectual disability, or other serious psychiatric conditions that affect their ability to use mobile phones or computers were excluded. Caregivers were referred to as those who care for patients diagnosed with PD or dementia as formal (ie, paid) or informal (ie, unpaid) caregivers. HCPs included those who worked in the medical or social care of patients diagnosed with PD or dementia (eg, physiotherapists, physicians, and occupational therapists). Further details regarding eligibility criteria and recruitment are reported in the study protocol [24].

### Quantitative Data Collection and Analysis

In total, 2 anonymous web-based surveys were created in English through EUSurvey tool and were translated into the other 5 project languages (German, Italian, Portuguese, Romanian, and Spanish). Both surveys were available on the PROCare4Life official website [39], in the period between May 27, 2020, and July 31, 2020, along with a short explanation of the purpose of the surveys. In addition, the surveys were disseminated through consortium member databases, networks, and national patient associations. The questions were developed in collaboration with clinical partners and aimed to gather answers regarding the topics listed in Textbox 1.

A descriptive analysis of the quantitative data was applied, including descriptive statistics and frequencies. The 7-item abbreviated Zarit scale was analyzed using SPSS statistical software (version 27; IBM Corp).

**Topics covered by the web-based surveys.**

1. Demographic data
2. Disease-related symptoms, assessed through a list of formulated questions regularly used by one of the pilot centers in this study—Asociación Parkinson Madrid—in evaluating the disease symptoms and medication side effects. These questions were approved for their content by the Movement Disorders Study Group of the Spanish Society of Neurology [40]
3. Difficulties in activities of daily living, assessed through domains derived from the self-reported Barthel index [41], in addition to other domains related to difficulties moving around and accessing health care centers
4. Caregiver family burden, assessed through the 7-items abbreviated Zarit scale [42,43]
5. Assistive health technology experiences and acceptance, assessed through developed Likert-items questionnaires assessing the frequency of use and the importance of technology devices intended to be included in the PROCare4Life platform (eg, tablet or mobile app)
6. Expected benefits and desired features in the proposed PROCare4Life platform. On a Likert-items questionnaires, the key performance indicators to be achieved within the project (eg, feelings of safety, feelings of autonomy, perception of empowerment, improvement of social participation, mental condition, and physical condition) and the main functionalities to be included were assessed

### Qualitative Data Collection and Analysis

Owing to the explorative nature of the study, individual interviews and workshops with open-ended questions were conducted in the period between June and July 2020. Each interview lasted between 30 and 60 minutes, and the following three types of interviews were conducted:

1. Interview for patients covering the same topics as in the quantitative study and allowing more exploratory answers through open-ended questions
2. Interview for caregivers covering their working experience, in addition to the topics mentioned in the web-based survey
3. Interview for key stakeholders covering their opinions, experiences, and ideas about using integrated digital health care platforms in the management of older people with NDDs, in addition to strengths, weaknesses, and the possible ways to promote the proposed PROCare4Life platform from their point of view

Additionally, 4 workshops were conducted between July and August 2020, with a duration of approximately 2 hours each. They involved HCPs and covered topics related to their experiences with using integrated digital health care technologies in the care of people with NDDs, expected benefits, barriers, and their requirements regarding the properties of the proposed PROCare4Life platform. The workshops also included discussions about preliminary results from the web-based surveys, which allowed more interaction between the participants, aiming to enrich the data collected.

Owing to the pandemic situation in most of the European countries at the time of the study, this qualitative study was conducted on the web except for a few interviews, where participants requested a face-to-face interaction; in those cases, COVID-19 social distancing and safety measures were all considered.

Gathered data were recorded, transcribed verbatim, and translated into English. Thematic analysis [44,45] was applied, using MAXQDA software (version 20; VERBI GmbH). Following a deductive-inductive approach, a framework containing the key topics covered in the quantitative study was developed. In total, 2 researchers worked independently and performed a first round of identifying the relevant text and coding (the four-eyes principle). The developed framework was applied to the entire data set but considering that the qualitative study included all the target groups, unlike the quantitative study, the researchers conducted a second round of open coding to identify additional topics and subtopics. The identified initial codes were discussed in a workshop involving the aforementioned researchers along with another researcher; significant data overarching the key topics were also discussed and validated in an iterative manner (discussion<->modification) to develop themes and subthemes (STs). The results and illustrative quotes were discussed with a researcher from Asociación Parkinson Madrid until a consensus was reached and themes were finalized.

### Mixed Methods Analysis

**Figure 1:**
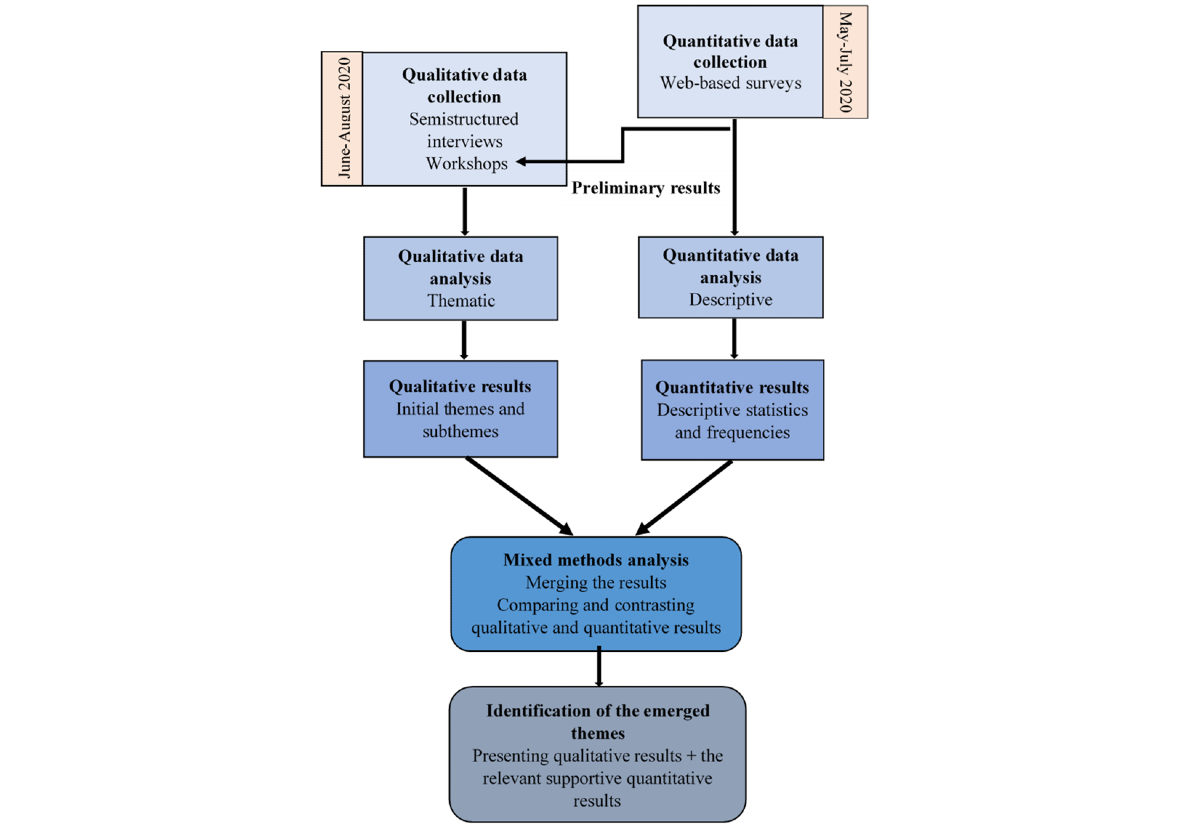
Mixed methods flowchart.

Both data types were analyzed separately. The identified initial qualitative themes and the main quantitative results were merged, aiming to combine the results and present them as the final emerged themes and STs (Figure 1). Although quantitative results provided numerical conclusions from our research and predicted outcomes about each theme, qualitative results allowed more comprehensive insights and in-depth knowledge from our participants about the same themes.

Because the topics in the surveys were also discussed during the interviews, the results were consistent, in particular those related to patients and caregivers. However, as the qualitative data were broader and involved all the participants, it included exclusive STs that had no corresponding supportive quantitative data. As these additional STs were considered important and provided a comprehensive reflection of the participants’ point of view, we included them in the mixed methods analysis as well.

Finally, 3 main emerged themes along with several STs were identified, which are presented in the *Results* section.

### Ethics Approval

The study protocol was approved by local ethical committees in Germany (number 020-37-MB), Italy (number 493-2020), Portugal (number 10-20), Romania (number 7/10.06.2020), and Spain (number 20/453-E). The organizations conducting this study established procedures for data protection management before the start of any processing of personal data, according to legal regulations and following good practices in research.

According to Good Clinical Practice and International Conference on Harmonization standards, once the study was fully explained, a written or digital informed consent was obtained from each participant before any study-related procedures.

There were no direct physical risks to the participants. Participation was entirely voluntary, and the participants had the right to withdraw from the study at any time, without giving reasons or experiencing any disadvantage. In case of withdrawal, no replacement was considered.

## Results

### Participants

Table 1 shows an overview of the participants in this study. Across study methods, countries, and target groups, a total of 217 participants were recruited. Overall, 72.4% (157/217) completed the 2 web-based surveys, distributed as 85 (54.1%) patients and 72 (45.9%) caregivers. The remaining 27.6% (60/157) of participants took part in the qualitative research: 20 (33.3%) HCPs were included in the workshops and 5 (8.3%) patients, 5 (8.3%) caregivers, and 30 (50%) key stakeholders took part in the semistructured interviews.

The characteristics of the end users are presented in Table 2. A total of 187 end users participated in the qualitative and quantitative strands of the study; most of them (71/187, 37.9%) were aged between 61 and 75 years, with more than half (112/187, 59.9%) being female. Most of the patients (64/90, 71%) who took part in this study were diagnosed with PD, and 96% (86/90) lived at home. More than one-third of them (32/90, 36%) rated their general health status in the past 4 weeks as fair. Most caregivers (68/77, 88%) were informal, and around half of them (38/77, 49%) lived with the patients they care for. In the workshops, HCPs from different specialties participated; however, 25% (5/20) were physiotherapists.

**Table 1:** Overview of the participants across study methods, countries, and target groups (N=217).

Country	Study method, n (%)												Total, n (%)
	Quantitative (n=157)		Qualitative (n=60)										
	Surveys		Interviews (n=40)							Workshops (n=20)			
	Patients (n=85)	CGs^a^ (n=72)	Patients (n=5)	CGs (n=5)	Media actors (n=6)	Academia (n=8)	Policy makers (n=8)	Market actors (n=8)	HCPs^b^ (n=20)				
Germany	3 (3.5)	13 (18)	1 (20)	1 (20)	2 (33.3)	2 (25)	3 (37.5)	4 (50)	5 (25)		34 (15.7)		
Italy	13 (15.3)	11 (15.3)	1 (20)	1 (20)	1 (16.6)	N/A^c^	2 (25)	2 (25)	3 (15)		34 (15.7)		
Portugal	27 (31.8)	8 (11.1)	1 (20)	1 (20)	2 (33.3)	4 (50)	2 (25)	2 (25)	7 (35)		54 (24.9)		
Romania	27 (31.8)	23 (32)	N/A	1 (20)	N/A	2 (25)	N/A	N/A	N/A		53 (24.4)		
Spain	14 (16.5)	17 (23.6)	2 (40)	1 (20)	1 (16.6)	N/A	1 (12.5)	N/A	5 (25)		41 (18.9)		
Others^d^	1 (1.2)	N/A	N/A	N/A	N/A	N/A	N/A	N/A	N/A		1 (0.4)		

^a^CG: caregiver.

^b^HCP: health care professional.

^c^N/A: not applicable.

^d^“Others” was one of the country choices listed in the web-based surveys, and the participants who answered with “others” were included in the data analysis.

**Table 2:** Characteristics of the end users (n=187).

Characteristics			End users, n (%)				Total, n (%)
			Patients (n=90)	Caregivers (n=77)	HCPs^a^ (n=20)		
**Age (years)**							
	<60		N/A^b^	44 (57.1)	20 (100)	64 (34.2)	
	61-75		46 (51.1)	25 (32.5)	N/A	71 (38)	
	>75		44 (48.9)	8 (10.4)	N/A	52 (27.8)	
**Sex**							
	Male		50 (55.6)	23 (29.9)	2 (10)	75 (40.1)	
	Female		40 (44.4)	54 (70.1)	18 (90)	112 (59.9)	
**Patients**							
	**Diagnosis**						
		Parkinson disease	64 (71.1)	N/A	N/A	N/A	
		Alzheimer disease	5 (5.6)	N/A	N/A	N/A	
		Other dementias	21 (23.3)	N/A	N/A	N/A	
	**Living situation**						
		At home	86 (95.6)	N/A	N/A	N/A	
		At home and temporarily at a day care center	2 (2.2)	N/A	N/A	N/A	
		At a residential center	1 (1.1)	N/A	N/A	N/A	
		Not reported	1 (1.1)	N/A	N/A	N/A	
	**General health status (4 weeks)**						
		Very poor	4 (4.4)	N/A	N/A	N/A	
		Poor	18 (20)	N/A	N/A	N/A	
		Fair	32 (35.6)	N/A	N/A	N/A	
		Good	24 (26.7)	N/A	N/A	N/A	
		Very good	7 (7.8)	N/A	N/A	N/A	
		Not reported	5 (5.6)	N/A	N/A	N/A	
**Caregivers**							
	**Type of caregiver**						
		Informal	N/A	68 (88.3)	N/A	N/A	
		Formal	N/A	7 (9.1)	N/A	N/A	
		Not reported	N/A	2 (2.6)	N/A	N/A	
	**Living with the person you care for**						
		Yes	N/A	38 (49.3)	N/A	N/A	
		No	N/A	28 (36.4)	N/A	N/A	
		Partially	N/A	8 (10.4)	N/A	N/A	
		Not reported	N/A	3 (3.9)	N/A	N/A	
**HCPs**							
	**Specialty**						
		Neurologists	N/A	N/A	1 (5)	N/A	
		Nurses	N/A	N/A	3 (15)	N/A	
		Psychologists	N/A	N/A	3 (15)	N/A	
		Physiotherapists	N/A	N/A	5 (25)	N/A	
		Speech therapists	N/A	N/A	3 (15)	N/A	
		Music therapists	N/A	N/A	1 (5)	N/A	
		Social workers	N/A	N/A	2 (10)	N/A	
		Educational trainers	N/A	N/A	2 (10)	N/A	

^a^HCP: health care professional.

^b^N/A: not applicable.

### Emerged Themes

#### Overview

**Figure 2:**
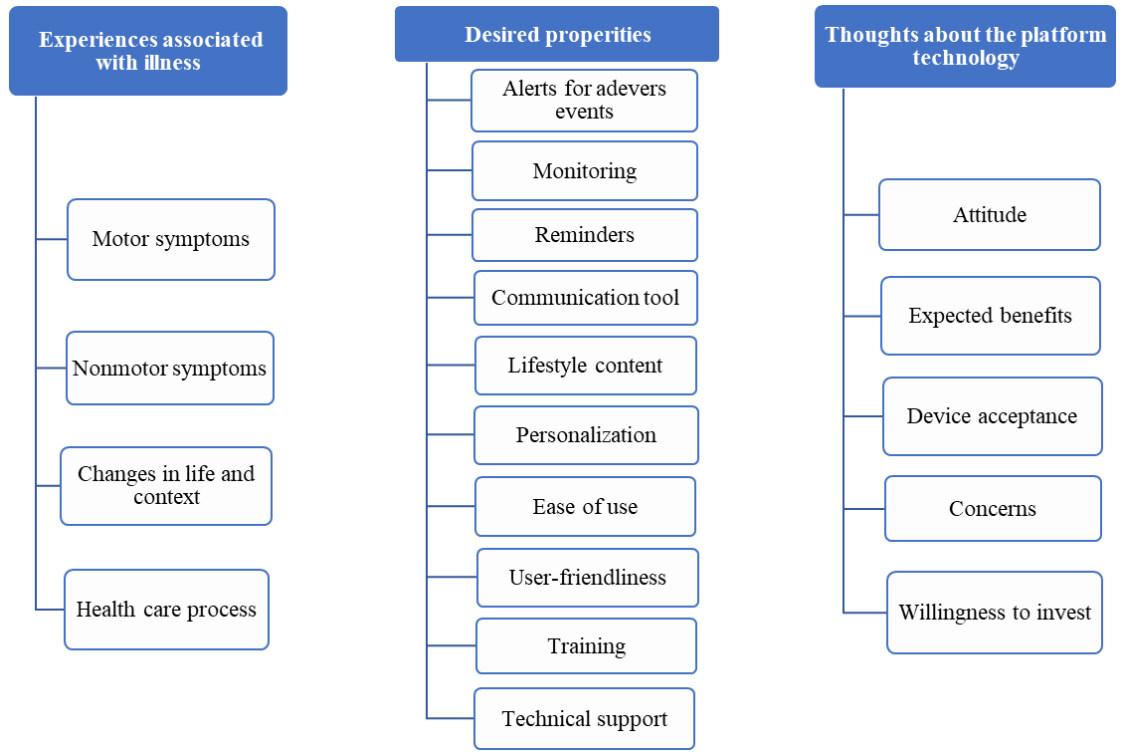
Overview of the emerged themes and subthemes.

In this section, we present the 3 emerged themes developed based on merging and interpreting the qualitative and quantitative data, namely experiences associated with illness (theme 1), thoughts about the platform technology (theme 2), and desired properties (theme 3). For every emerged theme, different STs were identified (Figure 2). We first present the detailed qualitative findings for each ST, followed by the relevant supportive quantitative findings. In addition, Table 3 illustrates a summary of the mixed methods analysis for all 3 themes and STs.

**Table 3:** Mixed methods analysis (some ratios are approximated).

T^a^ and ST^b^			Qualitative data codes		Supportive quantitative results (survey results)
**T1: experiences associated with illness**					
	ST1.1: motor symptoms	Stiffness Loss of balance Frequent falls and injuries Feeling of insecurity and disability Limited mobility, and physical limitations		Most reported motor symptoms: Stiffness was reported by 78% (66/85) of the patients and 83% (60/72) of the caregivers Loss of balance was reported by 66% (56/85) of the caregivers and 83% (60/72) of the caregivers	
	ST1.2: nonmotor symptoms	Concentration problems Memory problems Difficulties in communication Risks of medication misuse Missing meals Disorientation		Most reported nonmotor symptom is as follows: Difficulties in concentration was reported by 58% (49/85) of the patients and 63% (45/72) of the caregivers	
	ST1.3: changes in life and context	Difficulties in ADL^c^ Daily struggles Patients need support in everything Feelings of isolation Distressed and overworked caregivers Coping strategies		Difficulties in performing ADL and caregiver burden scale are as follows: Patients had difficulties in performing 15 of the ADL listed in the survey, as reported by >50% (38/72) of the caregivers In the 7-item abbreviated Zarit scale, 57% (41/72) of the caregivers reported to have family burden	
	ST1.4: health care process	Complex health care process Shortage in the number of HCPs^d^ Long waiting time for patients Limited time offered by HCPs Difficulties in accessing health care sites by the patients		Reported difficulties in performing the following activities: Difficulties in accessing therapy sites was reported by 43% (37/85) of the patients and 79% (57/72) of the caregivers Difficulties in accessing rehab sites was reported by 43% (37/85) of the patients and 68% (49/72) of the caregivers	
**T2: thoughts about the platform technology**					
	ST2.1: attitude	Positive: supportive, needed, loving it, and specifically needed and accepted at pandemic situation Negative: complicated, stressful, difficult, and not for everyone Aid and not replacement		N/A^e^	
	ST2.2: device acceptance	Smartphones, wearables, and tablets are highly accepted Smart television is complicated Cameras are invasive		Most accepted devices are as follows: Wearables: 57% (48/85) of the patients would like or love to use it Smartphones and tablets: 69% (50/72) of the caregivers would like or love to use it Least accepted devices are as follows: Cameras: 33% (28/85) of the patients and 31% (22/72) of the caregivers would like or love to use it	
	ST2.3: expected benefits	Improving communication between patients, caregivers, and HCPs Supporting the integrated care approach Improving work efficiency Supporting patient independency, caregiver engagement, and relationship between patients and HCPs Improving the health care process		The following expected benefits were agreed upon by >50% (43/85) of the patients and around 50% (34/72) caregivers: Increase the feelings of safety or autonomy of patients in their homes Improve the patient’s mental or physical condition Increase the patient’s perception of empowerment Improve the patient’s social participation Valid tool to respond to your needs	
	ST2.4: concerns	Privacy, data protection Costs Handling abilities specially by the patients		N/A	
	ST2.5: willingness to invest	Financial investment needs organizational support Individuals pay when there are benefits End users are willing to invest time and effort to learn using the platform		Willingness to pay: 51% (43/85) of the patients and 49% (35/72) of the caregivers were not sure if they would pay for the platform	
**T3: desired properties**					
	ST3.1: alerts for adverse events	Detecting and recording hazardous situations Relief for caregivers Need a supportive infrastructure		Alerts for adverse events was reported as a desired functionality by 80% (68/85) of the patients and 90% (65/72) of the caregivers	
	ST3.2: monitoring	Continuous monitoring of vital signs Health status measures Movement and gait changes in patients with Parkinson disease Sleep disorders Symptoms evolution Medication side effects Monitoring in real time		Monitoring tool was reported as a desired functionality by 85% (72/85) of the patients and 82% (59/72) of the caregivers Real-time information was reported as a desired feature by 80% (68/85) of the patients and 86% (62/72) of the caregivers	
	ST3.3: communication tool	Communication with HCPs Communication with peers Chat tool Get information at home Digital interviews, interventions, and follow-up sessions The need for in-person contact		Communication tool was reported as a desired functionality by 79% (67/85) of the patients and 88% (63/72) of the caregivers	
	ST3.4: reminders	Appointments Medications Drinking and mealtime reminders Relief for families		Reminders and a tool to organize appointments was reported as a desired functionality by 67% (57/85) of the patients and 75% (54/72) of the caregivers	
	ST3.5: lifestyle content	The need for PA^f^ and nutrition recommendations Cognitive games		Social networking tool was reported as a desired functionality by 60% (51/85) of the patients and 68% (49/72) of the caregivers Lifestyle recommendations (nutrition and PA) was reported as a desired functionality by 75% (64/85) of the patients and 79% (57/72) of the caregivers	
	ST3.6: ease of use	Simple, passive platform Low interaction Easily retrievable information No overwhelming emails and requests		Easy to set up and start platform was reported as a desired feature by 75% (64/85) of the patients and 89% (52/72) of the caregivers^g^ Few steps to get the functionality you want was reported as a desired feature by 79% (67/85) of the patients and 88% (63/72) of the caregivers^g^	
	ST3.7: personalization	Platform that is adapted to users’ skills and cognitive abilities Platform that considers the different target users Platform that provides relevant information.		Platform adapted to users’ skills was reported as desired feature by 74% (63/85) of the patients and 83% (60/72) of the caregivers^g^	
	ST3.8: user-friendliness	Comfortable wearables Predefined layout Less text More graphs and diagrams		Comfortable wearables was reported as a desired feature by 72% (61/85) of the patients and 81% (58/72) of the caregivers^g^	
	ST3.9: training	Provide training for end users Supportive manuals One-on-one training sessions		N/A	
	ST3.10: technical support	Provide supportive infrastructure (eg, networks, WiFi, and 5G) Hotline for technical support Automatic updates and backups		N/A	

^a^T: theme.

^b^ST: subtheme.

^c^ADL: activities of daily life.

^d^HCP: health care professional.

^e^N/A: not applicable.

^f^PA: physical activity.

^g^Different features as listed in the surveys with answers of very important or important (%).

#### Theme 1: Experiences Associated With Illness

In this theme, we present what the participants expressed regarding the NDD symptoms, how the illness affected their everyday lives, and the difficulties encountered within the health care services.

##### ST1.1: Motor Symptoms

Among the different motor symptoms associated with NDDs, stiffness and loss of balance were frequently mentioned. Most patients and caregivers expressed their concerns regarding the consequences of motor symptoms, such as frequent falls and injuries:

It is normal, but I am concerned because consequences can be severe. Fear of consequential damage (broken bone, etc.)

Caregiver, Germany

Furthermore, patients’ mobility becomes limited, and moving around becomes problematic and physically demanding, in particular for patients with PD:

At least from what I have seen with Parkinson patients, having to move and go somewhere is very physically demanding.

Academia 1, Germany

The consequences were not only physical; patients also explained that because of motor symptoms, they feel hindered and insecure:

I couldn’t move myself without being looked at and wobbled around. He is drunk or something, right? That hindered me a lot. I don’t want to say handicapped, but very upset...That’s what bothers me the most. Being insecure, it’s so bad that I feel really bad.

Patient with OD, Germany

##### ST1.2: Nonmotor Symptoms

Nonmotor symptoms represent another clinical spectrum of NDDs. For most patients and caregivers, disorientation and difficulties in concentration are worrisome, as patients can get lost:

The other day I got lost while I was going to the association. I went through a different street and suddenly, I did not know where I was.

Patient 1 with PD, Spain

Notably, memory problems are the hardest to deal with:

The hardest thing is to deal with memory problems.

Caregiver, Spain

They mentioned how memory problems could induce other challenges, such as difficulties in communication, as patients can forget the topics they are discussing or fail to identify with whom they are talking:

Sometimes I can’t remember their names, and in the middle of talking, I just stop. Sometimes it happens that I don’t even remember what I wanted to say. I don’t know where it comes from but it happens. And it’s not good, and I’m a little worried.

Patient with OD, Germany

Furthermore, owing to memory issues, patients can experience risky situations, such as an overdose or underdose of their medications, as well as missing mealtimes:

She [the patient] would forget to eat, saying she’s full; I don’t leave medication within her reach as she would either not take it or take more than actually prescribed.

Caregiver, Romania

##### ST1.3: Changes in Life and Context

After being diagnosed with NDDs, both patients and caregivers reported feeling isolated. Although patients felt apathetic and sad at home, caregivers had to rearrange their daily routines to focus all their efforts on taking care of the patients. In fact, one of the caregivers described caring for patients with NDDs as caring for a grown-up child, as they need support in almost all their activities:

It’s complicated, emotionally and logistically. Since the diagnosis, I stopped what I used to do. I only leave the house for urgent things.

Caregiver, Portugal

In their attempts to live with the illness, both patients and caregivers reported some coping strategies. For example, one of the patients with PD mentioned engaging in sports and being more physically active:

Try and compensate for the effects of the disease, I do sport, games, computer activities, keep house accounts. I keep active in general and I practice sports in particular.

Patient 2 with PD, Spain

Another patient with PD reported making to-do and shopping lists, which can help organize daily tasks:

I make lists and lists about money, about other things.

Patient 1 with PD, Spain

Meanwhile, caregivers also need to cope and make symptom-specific adjustments, such as adjustments to the living place or nutritional considerations:

Of course, you try to make the most of it. So, for example, in the case of swallowing disorders, you look at whether you are thickening or purify the food, and I just see that it is nicely prepared...To reduce the risk of falls, for example I’ve taken a carpet out of the living room before to avoid a risk of falling. Such things. So, I’m just advising a lot. So, even if certain things are worrying, we still have to deal with them.

Caregiver, Germany

##### ST1.4: Health Care Process

The health care process for patients with NDDs usually involves >1 specialty and requires many visits. Considering their *motor symptoms*, going to all these visits is demanding for both patients and caregivers, particularly those who live in rural areas:

You have the foot care, the pedologists—rarely now, because they are all fully booked. And just this whole medical complex that works together. You still have one or the other family doctor who still makes home visits. And then it stops. Because occupational therapists, speech therapists are rather rare and difficult to get here in the countryside.

Caregiver, Germany

Other challenges regarding the health care process were reported, such as a poor physician-to-patient ratio. On one hand, this can be stressful for HCPs as they have to manage extra numbers:

Doctors have more patients than they can handle and are late in seeing patients.

Patient 1 with PD, Spain

On the other hand, patients have to wait longer to get their appointments:

Right now, in Spain for patients to be seen by a specialist health professional, they have a 6 months period wait, and to get a social worker appointment it’s more.

HCP, Spain

Patients are usually not satisfied with the time offered to them by HCPs:

I’d say that nursing hours are too short. This makes the patients very disappointed that they can’t even talk to them a little. And then they (patients) are very sad.

Patient with OD, Germany

##### Supportive Quantitative Results for Theme 1

In the web-based surveys, both patients and caregivers were asked to report about the symptoms experienced by patients and whether they were worried about them; the difficulties in performing activities of daily living (ADL); and family burden for caregivers.

Stiffness was the most experienced symptom as reported by both patients (66/85, 78%) and caregivers (60/72, 83%), whereas stumbles and falls were the most frequent symptoms patients expressed that they were worried about (19/33, 58%). Regarding the nonmotor symptoms, feeling sad was the most common symptom reported by patients (61/85, 72%), whereas feeling anxious or nervous was reported by 69% (50/72) of the caregivers. Difficulties in communication was reported by 55% (47/85) of the patients and 68% (49/72) of the caregivers.

As for ADL, dressing and undressing was the most difficult activity for the patients (46/85, 54%), whereas accessing therapy sites and moving outside the house was the most difficult for the patients as reported by 79% (56/72) of the caregivers.

On the basis of the results from the 7-item abbreviated Zarit scale, 57% (41/72) of the caregivers reported having family burden.

All the detailed results from the surveys regarding this theme are provided in the Multimedia Appendix 1.

#### Theme 2: Thoughts About the Platform Technology

In this theme, the opinions of the participants regarding the proposed platform technology were gathered, including their attitudes toward different aspects of the platform technology, device preferences, their expected benefits and main concerns, and their willingness to use it and pay for it.

##### ST2.1: Attitude

On being introduced to the concept of the PROCare4Life platform and its main objectives, the participants showed varied attitudes. Some were positive about the initial platform design; in fact, several patients and caregivers said they personally loved it. HCPs and key stakeholders found it to be helpful, interesting, and required in the health care process:

Not only interesting but also very much needed.

Media actor, Portugal

Notably, most of the participants referred to the COVID-19 pandemic and the subsequent lockdown in most of the European countries as a reason for the increased interest in digital integrated communication platforms in health care. Patients have become more flexible about using ICTs:

I would use a tablet, for example, if we have a pandemic or something like that.

Patient with OD, Germany

HCPs thought that PROCare4Life is needed to continue providing services to their patients in situations where access to health institutions and facilities was limited for emergencies only:

All this COVID 19 situation changed everybody’s perspective. Not being able to be with people but still wanting to care for them. If we could have a digital system that allowed us to monitor someone at a distance and that also allowed us to be in contact and interact with them, that would be very important.

Market actor 2, Portugal

Furthermore, the pandemic was thought to be a catalyst in developing the market of digital integrated care platforms.

A negative attitude was also reported: one of the patients referred to old age as a challenge for accepting technological devices and benefiting from it. Some caregivers found the platform complicated, in particular for patients with advanced dementia, when the abilities to use any technical device become questionable:

This only works if there is no disease that does not affect it. With advanced dementia, the use of such devices no longer works at all.

Caregiver, Germany

In addition, a negative attitude from some HCPs was based on the opinion that such a platform could be an additional burden to their work, both time-consuming and stressful:

It can cause some stress to the team since there's an additional pressure and responsibilities.

HCP, Portugal

Finally, participants pointed out that health care technological solutions such as PROCare4Life should be only a support and not a replacement for physical contact and in-person interaction:

Technology can be considered a support and an aid, but not a replacement.

HCP, Italy

##### ST2.2: Device Acceptance

The initial design of the platform and different devices to be integrated were explained. Most patients and caregivers preferred using wearables and smartphones:

With an explanation and knowing the objective. Yes, she loves wearing devices and wanted an Android smartphone, she wears tele-assistance and likes it.

Caregiver, Spain

However, during the workshop in Germany, some HCPs preferred tablets over smartphones, referring to the negative experience they had regarding the smartphones’ usability:

With smartphones it was certainly the case-so the feedback that it was difficult to use because people didn’t understand it well and the volume was so low.

HCP, Germany

In addition, including stationary devices was seen as helpful, as it can ensure continuous monitoring of the patients in case they forgot to wear their wearables:

The strength of having sensors at home is that if they have a wearable system, people might forget to put it on. Fixed sensors will be better, because they will always be present.

Media actor, Portugal

Conversely, some devices were less accepted, such as a smart television, which was thought to be difficult for patients with dementia, and cameras, owing to data protection and privacy intrusion worries:

Dealing with this [smart TV] is difficult...I would prefer not to use it [cameras], even if it doesn’t record images, I see a privacy problem, although it might make sense. But it’s data protection difficult and I don’t know if I want to be monitored by technology all day long.

Caregiver, Germany

##### ST2.3: Expected Benefits

The participants expected several benefits when using the platform. Improvements in communication among patients, caregivers, and HCPs, as well as among HCPs, was frequently mentioned:

Communication between specialists could probably be better, and between the specialists and the patients also.

Caregiver, Spain

This, in turn, enables information sharing among all the stakeholders involved in the health care process and supports the multidisciplinary approach for patients:

It is good for both parties. For example, if someone comes to the hospital, there would be direct information about that person. Then this saves them from repeating their medical history, especially if this does not work well anymore due to an illness. The same is the case with institutions. If I imagine from my professional field in the rehabilitation clinic to have this possibility and to be able to access the information directly, that would be very practical. After all, you want to provide the best possible care for people. By the bundled information you would have a good impression of the person and then you can give more individual advice regarding the future of the patients.

Market actor 1, Germany

For health care teams, the PROCare4Life platform was thought to help saving time and effort per patient, reduce the workload on the nursing staff, and subsequently improve the overall work efficiency:

I think, that I would benefit from PC4L in terms of working efficiency, saving time devoted for each patient and reduce the reachability time.

HCP, Italy

On an individual level, the platform was seen to help patients to live independently at home:

People can remain as long as possible in their own coziness, while they are still independent as long as possible.

HCP, Germany

It can ensure the engagement of caregivers in the health process and improve the relationship between patients and HCPs:

A positive aspect is the involvement of the caregiver, that usually is unfairly underestimated [...] enhancing the relationship of trust between patient and health professionals.

HCP, Italy

##### ST2.4: Concerns

Most of the concerns expressed by the participants regarding the proposed platform were related to privacy and data protection. Owing to the nature of such platforms that require sharing personal data, explaining everything to the user and obtaining their consent were considered a must. Meanwhile, concerns related to the security of the platform and the protection measures followed to secure the data were mentioned:

How secure is all this? So how secure is this server? So that’s what I always think. It is also very sensitive data. And we know: data protection, hackers—a lot can happen and you have to be aware of that.

HCP, Germany

Costs and the price of the platform were seen as a typical barrier not only for PROCare4Life but also for any digital health care solution:

The typical barrier I would say it is the price that it takes to be implemented.

Media actor, Italy

Finally, the questionable abilities of the patients to handle the proposed platform was reported as a concern:

I don’t think patients can handle it. They don’t know the technology well.

Caregiver, Germany

##### ST2.5: Willingness to Invest

Regarding the willingness to invest in the proposed platform, participants mainly commented on 3 areas of investment (financial, time, and effort). Most patients and caregivers were unsure about investing money to pay for this platform. Patients mostly wanted to see the benefits they would get from the platform before deciding, whereas formal caregivers thought that the platform should be financed by patients, their relatives, or health insurance authorities:

Depends on the perspective. I as a nurse no. This is what relatives and patients should pay for. Or actually the health insurance companies.

Caregiver, Germany

On the other hand, HCPs reported that health institutions would pay for such platforms if they are beneficial:

Yes, if it’s something new and that we can all benefit from that, we think our organization would pay for that.

HCP, Portugal

Another HCP suggested the platform to be financed by a third party or providing it as a rent service:

You think about who has to pay for a platform like that, you can simply offer it as a service. That can be financed by a project, or any other financing possibility than the user. Maybe in a rent form, I don’t know, 60 or even 300 Euros per month. As long as its value is favorable.

HCP, Germany

Regarding the time and effort to learn, the end users were more willing to invest in the proposed platform, stating that it pays off eventually:

It takes an initial time to get used to all the tools but it pays off in long-term.

HCP, Portugal

##### Supportive Quantitative Results for Theme 2

Questions related to device acceptance, expected benefits, and willingness to pay for the proposed platform were asked in the web-based surveys.

Patients and caregivers were asked about their acceptance for several technological devices that are thought to be included in the platform. On a Likert item questionnaire, wearable devices were highly accepted among patients; in fact, 57% (48/85) of them would like or love to use it, whereas mobile or tablet was more accepted by caregivers (50/72, 69%). Cameras were the least accepted, as only 33% (28/85) of the patients and 31% (22/72) of caregivers would like or love to use them.

Among a list of different expected benefits from the proposed platform, increasing feelings of safety or autonomy of patients in their homes was the most agreed upon by both patients (66/85, 78%) and caregivers (50/72, 69%).

When asked about their willingness to pay for the proposed platform, around half of the patients (43/85, 51%) and caregivers (35/72, 49%) answered “I don’t know.”

More detailed quantitative findings can be found in the Multimedia Appendix 2.

#### Theme 3: Desired Platform Properties and Supportive Measures

This theme reports about participants’ answers regarding the needed features and supportive functionalities to be included in PROCare4Life.

##### ST3.1: Alerts for Adverse Events

Some symptoms related to NDDs can appear suddenly. Including a function that detects adverse events and informs a responsible party about them was thought to be useful and a relief for patients and caregivers:

It [adverse events alert] would be very useful. So that I know if there’s something wrong with her and what it could be. It would leave me in peace.

Caregiver, Spain

Meanwhile, adverse event alerts support HCPs in detecting the daily irregularities or the symptom-induced hazardous situations of the patients and reporting them to the health care team:

A tool to measure, record and analyze on/off stages, swallowing, activities of daily living, falls, dangerous behavior, quality of sleep. With the possibility of reporting the adverse effects to the nurse.

HCP, Spain

However, one of the caregivers stated that for adverse event alert to be effective, supportive infrastructure is needed:

We often get calls that a patient in a city several hundred kilometers away. And we have nothing to do with that. It just makes us crazy and doesn’t help people. The infrastructure for this has not yet been properly developed.

Caregiver, Germany

##### ST3.2: Monitoring

Having a tool that monitors the patients’ health status was seen as very useful by most of the participants, as it provides continuous and objective information about the patients. It can be used to monitor the patient’s vital signs and other health-related measures:

So these systems could be used for monitoring pulse and sugar status and vitamin status and nutritional status and exercise status.

Market actor 2, Germany

HCPs were interested in monitoring the symptom evolution and the side effects of certain medications. Furthermore, monitoring the movement and gait patterns to detect any changes (ie, fall detection), particularly in patients with PD, was thought to be important:

One could also install sensors in the room and measure the movement patterns of the residents with Parkinson’s disease. This would also make it possible to recognize early on, for example, if the person has a certain movement pattern that he or she will soon fall. And warning signals can also be sent accordingly.

HCP, Germany

In addition, monitoring patients in real time was thought to be important:

The perspective of the information in a real moment it’s really important and the possibility to also maintain informed people of interest related to the caregivers or people who are monitoring them.

Academia 2, Germany

One of the caregivers stated that if patients were monitoring themselves all the time, without knowing how to interpret the values, it would be stressful for the patients:

If the patients are monitoring themselves, I find it horrible...And if patients always monitor themselves, I think they’re just afraid of the values and then it’s the bracelet or the fear of the values that stress the patients, not the disease itself.

Caregiver, Germany

This could mean that monitoring needs to be controlled or adapted to the users.

##### ST3.3: Communication Tools

Inclusion of communication tools that could work in different ways was frequently reported. First, the participants thought that a tool for communication that enables patients and caregivers to ask questions regarding their medications or issuing health reports and sick leave without in-person appointments with HCPs would be useful:

For example, information about drugs contraindicated in the disease or on aspects such as a sick leave, some simple information that would avoid me having to go to my health center.

Patient 1 with PD, Spain

In addition, HCPs can launch digital interviews and interventions, for instance, physiotherapy sessions that could be held on the web when the patients are not able to personally visit the health care institutions:

A tool that would allow video calls: Digital interviews, interventions.

HCP, Spain

Follow-up sessions could also be held through such tools:

For some phases video calls can be useful, I don’t know about therapy but I can imagine that you can have it where you meet physically but also have check-up during the week over a video call.

Academia 1, Germany

Second, one of the HCPs thought that a communication tool that facilitates interaction among HCPs from different specialties regarding their patients’ health conditions and treatments would be useful:

It would be great to have a two-way access and communication service with other professionals.

HCP, Spain

Another way for communication was suggested by one of the caregivers, which is implementing a chat tool or system where different caregivers and involved persons could share knowledge and interact:

A chat system! So that either a caregiver or qualified staff could answer questions, I mean, it should somehow serve as an interactive chat among all users, or a solution should be sought somehow; I mean, if I need to pose a question, it should pop up, as it would with a forum, and anyone should be able to answer that question, that is, if the site developer is not available right there and then, if someone else is nevertheless available to talk to me and share their opinion, then from the opinions of two or three participants I might extract a conclusion guiding me this or that way.

Caregiver, Romania

However, some of the local organizations participating in this project already have their own internal communication channels using WhatsApp or Facebook, through which they can interact with workers in their organization or organize some social events with their peers:

I am already in the WhatsApp group of the Parkinson sports team and also in the Facebook Parkinson care group.

Patient 1 with PD, Spain

Another point that a communication tool within the platform should not replace in-person communication, particularly in the case of dementia where personal contact is crucial, was highlighted:

I think with older people and with people with dementia is still, I think the personal conversation is still more important, maybe people with dementia don’t understand that it goes over a screen.

HCP, Germany

##### ST3.4: Reminders

With the memory problems associated with NDDs, integrating reminders that inform the patients of what they have done or suggest what they should do was frequently reported in the interviews for providing support to the caregivers and patients’ families:

The relatives are also relieved by this. Because they also think a lot for the patients. And when they no longer have to take over this reminder function, they are also relieved.

HCP, Germany

From the patients’ and caregivers’ perspectives, reminders can be sent for medication and mealtimes:

Maybe a device to tell her, to remind her of medication or meal hours. Or one that reminds me of all this when I’ve got my hands full at work, so that I can then give her reminder calls myself. For no matter how many memos I make, I get buried in my work and forget about them

Caregiver, Romania

For HCPs, reminders can help patients remember their medical appointments and ensure that their daily water intake is consumed:

A memory of drinking. Or we do—today at 11:00 o'clock–10 minutes of gymnastics.

HCP, Germany

##### ST3.5: Lifestyle Content

Inclusion of some support related to the lifestyle activities of both patients and caregivers was mentioned by the participants of this study. The areas of support reported were nutrition and physical activity:

Better implement an online nutrition program. I would always try to add sports and movement offers. Just as well as cultural aspects.

Academia 2, Germany

Sociocultural events and cognitive games were also preferred:

Yes, I would love to use it [games].

Patient 1 with PD, Spain

##### ST3.6: Ease of Use

The proposed platform needs to be simple and practical as stated by one of the patients with PD:

For me it is important that the system is practical and as simple as possible.

Patient 2 with PD, Spain

In the workshops, one of the HCPs mentioned that it is even better if the platform is passive, considering the cognitive abilities of the patients with NDDs:

In the case of people with cognitive impairment, it will also be important that the technology is not only easy but even passive.

HCP, Spain

Different criteria for ease of use were reported by all the participants, such as low interaction with the platform, easy navigation of the platform and retrieval of information, and fewer approvals and requests for setting up the platform.

One of the key stakeholders, who is also a clinical professional, stated that for clinical professionals, the easier the platform, the more it will be accepted and used:

For us as clinical practitioners I think that must be a solution where we can easily access the information and the information must be as simple as we can get. So, I think we need to have something that we don’t need to learn to use it, or at least if we have to, just learn a minimum of sets to use it because if it’s complex system, I think we are going to let it go. The easier to use, the easier it will be.

Academia 4, Portugal

##### ST3.7: Personalization

A relevant aspect of the platform is the ability to be customized to fit the users’ needs and preferences, especially those needs related to the cognitive problems of the patients:

Adapted to the needs of the person according to his/her cognitive state and preferences.

HCP, Spain

Personalization can be achieved by adapting the platform’s tools according to the target group’s needs. “*Reminders*” was highlighted as one of the desired tools. For example, a water intake reminder can be developed, which is important for older people with dementia who often forget to drink hydrating fluids:

People with dementia often have the problem that they do not drink enough. And maybe you could install a water dispenser for them.

HCP, Germany

It is important that the platform considers the various needs among different end user groups and that it offers flexibility of its functions so that the end users can adjust it according to their interests and needs:

Also, the personalization...not only by the professional but also by other users.

HCP, Spain

I think this is something individual. It’s not something you get off the shelf but like with the apps you can buy when you need something. That you can expand the system depending on the degree of illness you have or the need you have.

Market actor 2, Germany

Furthermore, the platform can support HCPs in personalizing their treatment plans when disease-specific information about the patients is available:

In my case as a physiotherapist, I would like to have access to information about habitual displacements or activities in which the person presents motor difficulty, to be able to focus the treatment towards a more functional objective. In this way we would achieve more personalized treatments.

HCP, Spain

##### ST3.8: User-friendliness

For the platform to be user-friendly, patients would like to have comfortable wearables that do not irritate their skin and familiar sensors that do not cause them anxiety:

Very important that it [wearables] doesn’t irritate.

Caregiver, Spain

For the HCPs, the user-friendly criteria were that the platform layout is predefined, has less text presentation, and focuses more on visualizing the information using symbols and graphs:

No text. I think most people don’t like to read text...I would like to see this in a traffic light system. So that the green area is everything ok, everything is good, with red something has to be done.

HCP, Germany

##### ST3.9: Training

HCPs and other key stakeholders pointed out the need for the end users to be trained on how to use the platform. This includes educating them about the different devices and providing training sessions on their use. Furthermore, training should not only rely on manuals but also provide some interactive training sessions:

I can learn it myself, but it is difficult for me to learn new programs by reading manual. As far as such new program I need someone who sits next to me and introduces me to the program.

Market actor 1, Germany

##### ST3.10: Technical Support

With a digital platform, technical issues can always arise. Therefore, the presence of technical support was seen as a need by HCPs and other key stakeholders. This support can be in the form of automatic backups of the stored information or as supportive infrastructure and networking:

If the system fails, having an automatic backup to prevent loss of information or any delays on the reports.

HCP, Portugal

In addition, a service hotline to report urgent technical issues is required:

A service hotline would also make sense. If problems arise, it is important to reach someone.

HCP, Germany

##### Supportive Quantitative Results for Theme 3

The web-based surveys included questions about several features and functionalities to be included in the platform. On a 3-point Likert-items questionnaire, a tool to monitor symptoms and activities was the highest-rated functionality, as 85% (72/85) of the patients found it very useful or useful. Most caregivers (65/72, 90%) found that a tool to detect adverse events, unusual activities, or movements was useful.

In addition, when asked about the importance of different features of the platform, 80% (68/85) of the patients reported the real-time information feature as very important, whereas approximately 89% (64/72) of the caregivers reported an easy-to-set-up platform as very important. Detailed results from the surveys regarding this theme are provided in the Multimedia Appendix 3.

## Discussion

### Principal Findings

This study aimed to identify the needs and requirements for an ICT-based integrated care platform in supporting its potential end users and other stakeholders involved in the health care process of NDDs by exploring the participants’ opinions regarding the health care process and digital health care solutions. Findings on experiences associated with NDD symptoms, the challenges faced by all the potential end users in health care services, and the inevitable changes to life and its context have been well documented [2,3]. This is perhaps why participants were so forthcoming with wanting to share their experience in the hope of a supportive technology solution.

Although both positive and negative attitudes of the participants toward PROCare4Life were explored, the participants expected several benefits when using the platform. These varied between supporting the patients’ empowerment and independence, increasing caregivers’ and HCPs’ work efficiency, and ultimately improving health care services. Notably, influenced by the COVID-19 pandemic, most participants referred to the platform as a need, with most of them being willing to use it. This finding is of particular importance, as older people with chronic illnesses have been identified as a vulnerable group, who require special consideration for encouraging them to use ICT in health care during pandemic times [46].

Most of the desired functionalities reported here confirm findings from previous research, such as the need for medication intake reminders, monitoring, and communication tools [32,47]. We provide more insights into the specific needs related to these functionalities, such as the patients’ constant need for meals, water intake, and appointment reminders. In addition, a monitoring functionality that provides objective data in real time for both caregivers and HCPs was reported, with emphasis on monitoring motor symptoms and gait patterns and installing a fall detection system. A communication tool was valued by all the potential end users, believing that it improves the relationship among patients, caregivers, and HCPs and facilitates the overall health care process. It is known that ICTs are useful in reducing social isolation and providing opportunities for older adults to keep in touch with the outside world [28,48,49]. Therefore, implementing a communication tool could alleviate the isolation and feeling of distress reported by the patients and caregivers in this study. Furthermore, as stated by one of the caregivers, a chat tool facilities interaction between peers and knowledge exchange among all the parties involved in the care process.

Another important finding was the need for personalization, which stems from the individuality of each user, including disease severity, experiences, and preferences. As the saying “One size does not fit all” goes, as this platform targets different end user groups, a flexible design should be considered. In addition, with the progressive nature of NDDs, there is a need to offer tailored functionalities that match the stage of the disease and patients’ abilities. Having too many functionalities and options might cause confusion for the end users, particularly for patients and caregivers. Similar to what has been reported by Boman et al [33], some of our participants pointed out the need to provide >1 version or package of the platform to facilitate the customization of the product.

The diversity of needs, along with a few contradictions between the end user groups, was the main challenge in our work. Although patients and caregivers were concerned about using stationary devices and cameras, HCPs expressed that including these devices is important for ensuring the real-time monitoring functionality of the platform. In addition, the results show how end user groups tend to have different perspectives when identifying a specific property. For instance, although all the participants highlighted the need for the platform to be easy to use and user-friendly, both patients and caregivers viewed this as having familiar objects and comfortable wearables, whereas HCPs cared more for the visualization (ie, having a traffic color system) and the layout of the platform. Furthermore, HCPs emphasized that a platform that is easy to navigate through and retrieve information from is more likely to be used. It is known that simplicity, ease of use, and understandable features increase the possibility of older people with impaired cognitive abilities using digital devices independently and for a long term [29,33,50-54].

The need for providing education about the platform and training on the devices to be used were stated by HCPs and other key stakeholders. Therefore, in addition to patients, offering training opportunities for HCPs and caregivers is of great importance. Staff who are familiar with the platform, have experience, and are interested in using it play an important role in encouraging their patients to use it [55,56].

It is also noteworthy that potential end users found it difficult to decide whether they would pay for the PROCare4Life platform and by how much. Although HCPs referred to this point as one of the typical barriers for ICT health care platforms, they were more willing to pay compared with patients and caregivers and believed that their institutions would be willing too. Identical to what was reported by Contreras-Somoza et al [48], who studied the acceptability of an ICT device for older adults with mild cognitive impairment, formal caregivers in this study expected PROCare4Life to be financed by the patients themselves, relatives, or health insurance companies. Furthermore, the patients in our study needed to grasp the benefits of the platform first before deciding on paying for it. HCPs suggested providing renting offers of the platform to ease the cost burden for those who would like to use it, in case no other financing possibility was available. Therefore, there is a need for providing a better investment in digital solutions that support healthy and independent aging, which relies on collaboration between the government, organizations, and the private health sector [48,57].

When developing an ICT platform that supports all the parties involved in the care process of older people with NDDs, identifying the users’ needs, interests, and abilities is crucial [58], regardless of being challenged with the diversity of needs and different priorities of the participants in this study. Combining different views from different perspectives (eg, patients, caregivers, HCPs, and other stakeholders) is thought to prevent individual concerns such as privacy issues from becoming a barrier for using technology [59,60] and ultimately increase the acceptability of technology in health care [47].

In addition, our participants valued this study and appreciated the idea of trying to include them in the development process and understanding their needs:

The strongest point for me is doing this interview, to really start asking what do people need and want and do they think they can use it. I think that it relates directly to usability, and the user experience which is crucial. I worked together with therapists to develop a system back there, but no matter how good the system was, if it’s not being used, there is nothing you can do about it. So, I think what you are doing is very important for the acceptance of the people for this system.

Academic Researcher 1, Germany

For this purpose, encapsulating a UCD approach in the development stages ensures addressing the real needs and avoiding poor final acceptance. Indeed, the rationale behind UCD is that the “purpose of any design is to serve the user, not to use a specific technology or to be an elegant piece” [61].

### Implications on PROCare4Life

The data collected and knowledge gained from this study were transferred to the development and research team of PROCare4Life with the purpose of designing and redesigning the different services of the platform. What ultimately emerged from this initial phase of the project supported the efforts to raise awareness about the major areas of users’ needs, where technological aspects of the platform could be more valuable. The identified “desired properties” represent the main core of the final platform solution based on a realistic idea of the problem and a better vision of what to prioritize for each of the properties, providing an insight into how the scheme of operation of the system was to be shaped. In addition, the work on specifying areas of technical interest to users is an ongoing process that shall continue throughout the project pilots as new needs emerge followed by further technical developments.

Furthermore, this study identified several prerequisites for the acceptance of this platform, for instance, training and technical support. Therefore, training manuals for users containing instructions for the implementation and use of the system have been produced and will be continuously adapted and improved until the end of the project for personalized configuration along with more training opportunities, such as interactive sessions and e-learning, which are currently being assessed in the framework of the pilots. In addition, each clinical site was assigned a technical partner for support and guidance in the event of any technical difficulty.

Regarding privacy and protection in the use of cameras, the technical team worked on a code with the depth camera and the real-time software to avoid the storage of any patient’s images. In addition, a simplified illustrative video was distributed among the users, explaining how the images acquired by the camera are directly processed by a software that generates an output composed of an 18-point skeleton in combination with the depth information. Thus, no images are saved, avoiding privacy issues.

### Strengths and Limitations

Developing a successful product means that the needs of the target groups are included [62]. This research was based on a large sample of participants, including different perspectives presented by patients, caregivers, HCPs, and other related key stakeholders. This ensured that all views on different needs and challenges are considered during the development and future pilot phases. The inclusion of patients with different NDDs (ie, dementia and PD) and the multisite (ie, 5 different European countries) approach allow the findings to be generalized so that the PROCare4Life platform solution can be applied to other chronic diseases and facilitates the exploitability of the achieved results in the long run. Furthermore, the mixed methodology study enriched the comprehensive data that reflected the diverse needs of the participants.

One of the main challenges this study faced was the COVID-19 pandemic, which directly impacted the dynamics and schedule of the PROCare4Life project in its first months of development. Confinement and social distancing measures implemented by all member states and local authorities in the different European countries required an adaptation in approaching and recruiting the participants involved in the study. Most data were collected remotely through phone or digital interviews and on the web; however, a few meetings were held face to face. This rearrangement required extra work for the researchers involved in this study, ensuring that the prevention and control measures to avoid infections were strictly applied. Surely, this situation created some challenges and limited in-person contact with the participants. Nevertheless, this study was able to approach the intended target groups and numbers. In addition, the PROCare4Life approach to the current pandemic situation was continuously documented in clinical partners' countries to ensure standardized preventive measures. This exchange led to the publication of a COVID-19 protocol that shares the instructions and suggestions of the respective national and local health authorities, which is available on the web [63]. In addition, the awareness of the pandemic seemed to open perspectives to the usefulness of the platform (eg, ST2.3).

By the time of the study, the design of the functionalities in the mock-ups were not developed yet. Although this was expected, considering the aim of the study and the early stage of the development process, some of the participants, in particular patients, were not able to imagine the design of the platform. It has been pointed out in the literature that patients with declined cognitive ability might have difficulty in imagining the things they cannot see or articulating their perceptions of the device intended to be used [33-47]. Considering this, HCPs and caregivers who were familiar with the patients were included. Furthermore, the early involvement of users in the process ensures the suitability of the product for its intended target group and purpose [64].

Another limitation is that this study prioritized qualitative data, which could mean losing some important quantitative results, in particular those related to patients and caregivers. However, the study design allowed the sharing of the same topics in both study methods. During the merging of the results, all the supportive quantitative data were considered. Finally, there was an overrepresentation of patients with PD in the study, which might have caused bias in the results toward motor symptoms as well as the inability of the study to draw conclusions on the specific needs of patients with other NDDs. The reason could be that patients with PD, especially in the early stages of the disease, are less likely to experience cognitive decline, and therefore, they are more easily engaged in this kind of study where they have to sustain attention and communicate their opinions clearly. In addition, in the countries involved in this study, associations of patients with PD are very active and eager to become involved. Nevertheless, samples were free from selection bias and were naturalistic under the overall umbrella of NDDs. For future studies, there might be a need for such kind of platforms to be designed for each neurodegenerative condition according to its own peculiarities.

### Conclusions

In this study, the needs of all the parties involved in the health care process of NDDs regarding an ICT-based health care platform were explored. The pandemic situation highlighted opportunities for digitalization in health care. The mixed methods approach yielded mostly consistent results, which were in line with findings from the literature. The collected data were useful for the development of the PROCare4Life platform.

Although the combination and collection of features for diverse user groups are typical for integrated care platforms, this results in exponential complexity for designers, developers, and users. Contradicting opinions and several concerns in this study demonstrate that an integrated care platform should not promise too much for too many. Instead, selection, focus, and, sometimes, restriction to the essentials are necessary. Users and other stakeholders should be involved in these decisions.

## Appendix

Multimedia Appendix 1 Results from the web-based surveys corresponding to theme 1 “experiences associated with illness.”.

Multimedia Appendix 2 Results from the web-based surveys corresponding to theme 2 “thoughts about the platform technology.”.

Multimedia Appendix 3 Results from the web-based surveys corresponding to theme 3 “desired properties.”.

## Abbreviations

ADL: activities of daily living
HCP: health care professional
ICT: information and communication technology
NDD: neurodegenerative disease
OD: other dementias
PD: Parkinson disease
PROCare4Life: Personalized Integrated Care Promoting Quality of Life for Older People
ST: subtheme
T: theme
UCD: user-centered design

## References

[1] (2011). Global Health and Aging. World Health Organization.

[2] De Marchi F, Contaldi E, Magistrelli L, Cantello R, Comi C, Mazzini L (2021). Telehealth in neurodegenerative diseases: opportunities and challenges for patients and physicians. Brain Sci.

[3] Olazarán J, Muñiz R, Reisberg B, Peña-Casanova J, del Ser T, Cruz-Jentoft AJ, Serrano P, Navarro E, García de la Rocha ML, Frank A, Galiano M, Fernández-Bullido Y, Serra JA, González-Salvador MT, Sevilla C (2004). Benefits of cognitive-motor intervention in MCI and mild to moderate Alzheimer disease. Neurology.

[4] Scalvini S, Giordano A (2013). Heart failure. Optimal postdischarge management of chronic HF. Nat Rev Cardiol.

[5] Bernini S, Stasolla F, Panzarasa S, Quaglini S, Sinforiani E, Sandrini G, Vecchi T, Tassorelli C, Bottiroli S (2020). Cognitive telerehabilitation for older adults with neurodegenerative diseases in the COVID-19 era: a perspective study. Front Neurol.

[6] Jia J, Wei C, Chen S, Li F, Tang Y, Qin W, Zhao L, Jin H, Xu H, Wang F, Zhou A, Zuo X, Wu L, Han Y, Han Y, Huang L, Wang Q, Li D, Chu C, Shi L, Gong M, Du Y, Zhang J, Zhang J, Zhou C, Lv J, Lv Y, Xie H, Ji Y, Li F, Yu E, Luo B, Wang Y, Yang S, Qu Q, Guo Q, Liang F, Zhang J, Tan L, Shen L, Zhang K, Zhang J, Peng D, Tang M, Lv P, Fang B, Chu L, Jia L, Gauthier S (2018). The cost of Alzheimer's disease in China and re-estimation of costs worldwide. Alzheimers Dement.

[7] Rodríguez-Blázquez C, Forjaz MJ, Lizán L, Paz S, Martínez-Martín P (2015). Estimating the direct and indirect costs associated with Parkinson's disease. Expert Rev Pharmacoecon Outcomes Res.

[8] Oh J, An JW, Oh SI, Oh KW, Kim JA, Lee JS, Kim SH (2015). Socioeconomic costs of amyotrophic lateral sclerosis according to staging system. Amyotroph Lateral Scler Frontotemporal Degener.

[9] Wake E, Atkins H, Willock A, Hawkes A, Dawber J, Weir KA (2022). Telehealth in trauma: a scoping review. J Telemed Telecare.

[10] Broese van Groenou MI, De Boer A (2016). Providing informal care in a changing society. Eur J Ageing.

[11] Kuluski K, Ho JW, Hans PK, Nelson ML (2017). Community care for people with complex care needs: bridging the gap between health and social care. Int J Integr Care.

[12] Kirst M, Im J, Burns T, Baker GR, Goldhar J, O'Campo P, Wojtak A, Wodchis WP (2017). What works in implementation of integrated care programs for older adults with complex needs? A realist review. Int J Qual Health Care.

[13] (2017). INclusive INtroduction of INtegrated CAre (IN3CA). European Commission.

[14] (2012). The European Innovation Partnership on Active and Healthy Ageing - Integrated Care and Chronic Disease Management. European Commission.

[15] Carretero S (2015). Technology-enabled Services for Older People Living at Home Independently: Lessons for public long-term care authorities in the EU Member States. European Commission.

[16] Bennett B, McDonald F, Beattie E, Carney T, Freckelton I, White B, Willmott L (2017). Assistive technologies for people with dementia: ethical considerations. Bull World Health Organ.

[17] Fares N, Sherratt RS, Elhajj IH (2021). Directing and orienting ICT healthcare solutions to address the needs of the aging population. Healthcare (Basel).

[18] Rajagopalan R, Litvan I, Jung TP (2017). Fall prediction and prevention systems: recent trends, challenges, and future research directions. Sensors (Basel).

[19] Lindberg B, Nilsson C, Zotterman D, Söderberg S, Skär L (2013). Using information and communication technology in home care for communication between patients, family members, and healthcare professionals: a systematic review. Int J Telemed Appl.

[20] Guisado-Fernández E, Giunti G, Mackey LM, Blake C, Caulfield BM (2019). Factors influencing the adoption of smart health technologies for people with dementia and their informal caregivers: scoping review and design framework. JMIR Aging.

[21] American Psychiatric Association (2000). Diagnostic and statistical manual of mental disorders (DSM-IV). 4th edition.

[22] Diaz-Orueta U, Hopper L, Konstantinidis E (2020). Shaping technologies for older adults with and without dementia: reflections on ethics and preferences. Health Informatics J.

[23] Smith M, Saunders R, Stuckhardt L, McGinnis JM, Committee on the Learning Health Care System in America, Institute of Medicine (2013). Best Care at Lower Cost: The Path to Continuously Learning Health Care in America.

[24] Ahmed M, Marín M, Bouça-Machado R, How D, Judica E, Tropea P, Bentlage E, Brach M (2021). Investigating users' and other stakeholders' needs in the development of a personalized integrated care platform (PROCare4Life) for older people with dementia or Parkinson disease: protocol for a mixed methods study. JMIR Res Protoc.

[25] (2019). Ergonomics of Human-System Interaction -- Part 210: Human-Centred Design for Interactive Systems. International Organization for Standardization.

[26] Steele Gray C, Barnsley J, Gagnon D, Belzile L, Kenealy T, Shaw J, Sheridan N, Wankah Nji P, Wodchis WP (2018). Using information communication technology in models of integrated community-based primary health care: learning from the iCOACH case studies. Implement Sci.

[27] Maresova P, Krejcar O, Barakovic S, Barakovic Husic J, Lameski P, Zdravevski E, Chorbev I, Trajkovik V (2020). Health–related ICT solutions of smart environments for elderly–systematic review. IEEE Access.

[28] Meiland F, Innes A, Mountain G, Robinson L, van der Roest H, García-Casal JA, Gove D, Thyrian JR, Evans S, Dröes RM, Kelly F, Kurz A, Casey D, Szcześniak D, Dening T, Craven MP, Span M, Felzmann H, Tsolaki M, Franco-Martin M (2017). Technologies to support community-dwelling persons with dementia: a position paper on issues regarding development, usability, effectiveness and cost-effectiveness, deployment, and ethics. JMIR Rehabil Assist Technol.

[29] Vermeer Y, Higgs P, Charlesworth G (2019). What do we require from surveillance technology? A review of the needs of people with dementia and informal caregivers. J Rehabil Assist Technol Eng.

[30] van der Roest HG, Meiland FJ, Comijs HC, Derksen E, Jansen AP, van Hout HP, Jonker C, Dröes RM (2009). What do community-dwelling people with dementia need? A survey of those who are known to care and welfare services. Int Psychogeriatr.

[31] Miranda-Castillo C, Woods B, Orrell M (2013). The needs of people with dementia living at home from user, caregiver and professional perspectives: a cross-sectional survey. BMC Health Serv Res.

[32] Lauriks S, Reinersmann A, Van der Roest HG, Meiland FJ, Davies RJ, Moelaert F, Mulvenna MD, Nugent CD, Dröes RM (2007). Review of ICT-based services for identified unmet needs in people with dementia. Ageing Res Rev.

[33] Boman IL, Persson AC, Bartfai A (2016). First steps in designing an all-in-one ICT-based device for persons with cognitive impairment: evaluation of the first mock-up. BMC Geriatr.

[34] Nieuwboer A, De Weerdt W, Dom R, Lesaffre E (1998). A frequency and correlation analysis of motor deficits in Parkinson patients. Disabil Rehabil.

[35] Espay AJ, Bonato P, Nahab FB, Maetzler W, Dean JM, Klucken J, Eskofier BM, Merola A, Horak F, Lang AE, Reilmann R, Giuffrida J, Nieuwboer A, Horne M, Little MA, Litvan I, Simuni T, Dorsey ER, Burack MA, Kubota K, Kamondi A, Godinho C, Daneault J, Mitsi G, Krinke L, Hausdorff JM, Bloem BR, Papapetropoulos S, Movement Disorders Society Task Force on Technology (2016). Technology in Parkinson's disease: challenges and opportunities. Mov Disord.

[36] Setareh Forouzan A, Mahmoodi A, Jorjoran Shushtari Z, Salimi Y, Sajjadi H, Mahmoodi Z (2013). Perceived social support among people with physical disability. Iran Red Crescent Med J.

[37] Lundberg S (2014). The results from a two-year case study of an information and communication technology support system for family caregivers. Disabil Rehabil Assist Technol.

[38] Creswell JW, Plano Clark VL (2010). Designing and Conducting Mixed Methods Research. 2nd edition.

[39] PROCare4Life.

[40] Spanish Society of Neurology. Movement Disorders Study Group.

[41] Mahoney FI, Barthel DW (1965). Functional evaluation: the Barthel index. Md State Med J.

[42] Gort AM, March J, Gómez X, de Miguel M, Mazarico S, Ballesté J (2005). Short Zarit scale in palliative care. Med Clin (Barc).

[43] Gort AM, Mingot M, Gomez X, Soler T, Torres G, Sacristán O, Miguelsanz S, Nicolas F, Perez A, de Miguel M, Cabau J (2007). Use of the Zarit scale for assessing caregiver burden and collapse in caregiving at home in dementias. Int J Geriatr Psychiatry.

[44] Boyatzis RE (1998). Transforming Qualitative Information: Thematic Analysis and Code Development.

[45] Braun V, Clarke V (2006). Using thematic analysis in psychology. Qual Res Psychol.

[46] Bajpai N, Biberman J, Ye Y (2020). ICTs and Public Health in the Context of COVID-19. Center for Sustainable Development, Columbia University.

[47] Meiland FJ, Hattink BJ, Overmars-Marx T, de Boer ME, Jedlitschka A, Ebben PW, Stalpers-Croeze II, Flick S, van der Leeuw J, Karkowski IP, Dröes RM (2014). Participation of end users in the design of assistive technology for people with mild to severe cognitive problems; the European Rosetta project. Int Psychogeriatr.

[48] Contreras-Somoza LM, Irazoki E, Castilla D, Botella C, Toribio-Guzmán JM, Parra-Vidales E, Suso-Ribera C, Suárez-López P, Perea-Bartolomé MV, Franco-Martín MÁ (2020). Study on the acceptability of an ICT platform for older adults with mild cognitive impairment. J Med Syst.

[49] Chen YR, Schulz PJ (2016). The effect of information communication technology interventions on reducing social isolation in the elderly: a systematic review. J Med Internet Res.

[50] Cahill S, Begley E, Faulkner JP, Hagen I (2007). “It gives me a sense of independence” – findings from Ireland on the use and usefulness of assistive technology for people with dementia. Technol Disabil.

[51] Evald L (2015). Prospective memory rehabilitation using smartphones in patients with TBI: what do participants report?. Neuropsychol Rehabil.

[52] Arthanat S, Simmons CD, Favreau M (2012). Exploring occupational justice in consumer perspectives on assistive technology. Can J Occup Ther.

[53] Jamieson M, Cullen B, McGee-Lennon M, Brewster S, Evans JJ (2014). The efficacy of cognitive prosthetic technology for people with memory impairments: a systematic review and meta-analysis. Neuropsychol Rehabil.

[54] Kintsch A, DePaula R (2002). A framework for the adoption of assistive technology. University of Colorado at Boulder.

[55] Nordin S, Sturge J, Ayoub M, Jones A, McKee K, Dahlberg L, Meijering L, Elf M (2021). The role of information and communication technology (ICT) for older adults' decision-making related to health, and health and social care services in daily life-a scoping review. Int J Environ Res Public Health.

[56] Gil AP, Moniz AB, de São Jose J (2019). Technologies in care for older people - EPTA Report 2019. European Parliamentary Technology Assessment.

[57] (2020). Cuarto examen y evaluación de la aplicación del Plan de Acción Internacional de Madrid sobre el Envejecimiento: Impulsando la agenda de envejecimiento. United Nations.

[58] García-Betances RI, Cabrera-Umpiérrez MF, Ottaviano M, Pastorino M, Arredondo MT (2016). Parametric cognitive modeling of information and computer technology usage by people with aging- and disability-derived functional impairments. Sensors (Basel).

[59] van Boekel LC, Wouters EJ, Grimberg BM, van der Meer NJ, Luijkx KG (2019). Perspectives of stakeholders on technology use in the care of community-living older adults with dementia: a systematic literature review. Healthcare (Basel).

[60] Olsson A, Persson AC, Bartfai A, Boman IL (2018). Sensor technology more than a support. Scand J Occup Ther.

[61] Mubashra A (2021). User Centred Design: a new essential for effective designs for the physically challenged. Int J Innov Creativity Change.

[62] McCabe L, Innes A (2013). Supporting safe walking for people with dementia: user participation in the development of new technology. Gerontechnology.

[63] (2021). COVID-19 Protocol. PROCare4Life.

[64] Duarte J, Guerra A (2012). User-centered healthcare design. Procedia Comput Sci.

