# Supplementary material for: End Users’ and Other Stakeholders’ Needs and Requirements in the Development of a Personalized Integrated Care Platform (PROCare4Life) for Older People With Dementia or Parkinson Disease: Mixed Methods Study
Source: JMIR Form Res. 2022 Nov 30;6(11):e39199. doi: 10.2196/39199 (PMC9752454; doi:10.2196/39199)

(Appendix 1)

Results from the web-based surveys corresponding to theme 1 “Experiences associated with Illness”

a. Symptoms associated with NDDs→ Corresponding to ST1.1, ST1.2, and ST1.4

The following tables and figures represent answers from both patients and caregivers regarding symptoms frequency and them being worried about it.

Question No. 15: (patient’s version) Have you experienced any of the following symptoms in the past month? Indicate YES or NO.

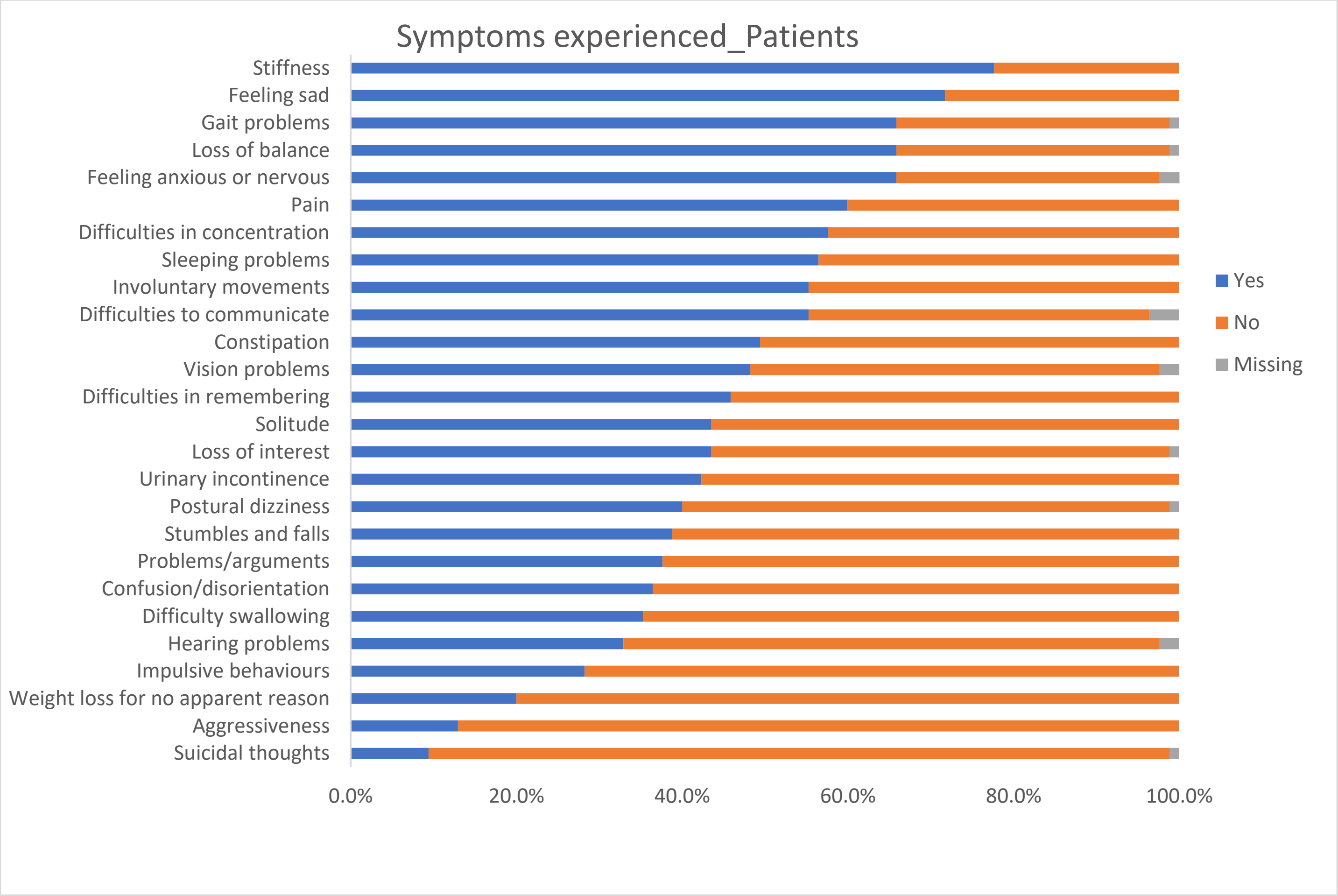

If you answer YES, please add on the right side of the table if you are worried about it?

Symptom	Patients` answers (n=85)			
	Yes %		No %	Missing %
		Worried about it* %		
**Stiffness**	77,6%	40,9%	22,4%	0,0%
**Feeling sad**	71,8%	26,2%	28,2%	0,0%
**Loss of balance**	65,9%	46,4%	32,9%	1,2%
**Gait problems**	65,9%	42,9%	32,9%	1,2%
**Feeling anxious or nervous**	65,9%	32,1%	31,8%	2,4%
**Pain**	60,0%	47,1%	40,0%	0,0%
**Difficulties in concentration**	57,7%	24,5%	42,4%	0,0%
**Sleeping problems**	56,5%	14,6%	43,5%	0,0%
**Involuntary movements**	55,3%	40,4%	44,7%	0,0%
**Difficulties to communicate**	55,3%	25,5%	41,2%	3,5%
**Constipation**	49,4%	33,3%	50,6%	0,0%
**Vision problems**	48,2%	34,2%	49,4%	2,4%
**Difficulties in remembering**	45,9%	20,5%	54,1%	0,0%
**Loss of interest**	43,5%	24,3%	55,3%	1,2%
**Solitude**	43,5%	16,2%	56,5%	0,0%
**Urinary incontinence**	42,4%	50,0%	57,6%	0,0%
**Postural dizziness**	40,0%	20,6%	58,8%	1,2%
**Stumbles and falls**	38,8%	57,6%	61,2%	0,0%
**Problems/arguments**	37,7%	28,1%	62,4%	0,0%
**Confusion/disorientation**	36,5%	16,1%	63,5%	0,0%
**Difficulty swallowing**	35,3%	40,0%	64,7%	0,0%
**Hearing problems**	32,9%	25,0%	64,7%	2,4%
**Impulsive behaviours**	28,2%	33,3%	71,8%	0,0%
**Weight loss for no apparent reason**	20,0%	52,9%	80,0%	0,0%
**Aggressiveness**	12,9%	2,4%	87,1%	0,0%
**Suicidal thoughts**	9,4%	25,0%	89,4%	1,2%

*worried about it: percentages are calculated from the total YES answers

<u>Question No. 18</u> (Caregivers version): Please, indicate which of the following symptoms the person you care for has difficulties. Indicate YES or NO. If you answer YES, please indicate on the right side of the table if you are worried about it?

Symptom	Caregivers` answers (n=72)			
	Yes%		No %	Missing %
		Worried about it* %		
Loss of balance	83,33%	51,67%	16,67%	0,00%
Stiffness	83,33%	43,33%	16,67%	0,00%
Stumbles and falls	73,61%	56,60%	26,39%	0,00%
Feeling anxious or nervous	69,01%	36,00%	29,57%	1,39%
Gait problems	68,06%	38,78%	30,56%	1,39%
Difficulties to communicate	68,06%	36,73%	31,94%	0,00%
Feeling sad	68,06%	34,69%	31,94%	0,00%
Confusion/disorientation	65,28%	36,17%	34,72%	0,00%
Difficulties in remembering	63,89%	30,43%	36,11%	0,00%
Difficulties in concentration	62,50%	24,44%	34,72%	2,78%
Pain	55,56%	42,50%	43,06%	1,39%
Sleeping problems	54,17%	25,64%	43,06%	2,78%
Vision problems	52,78%	36,84%	45,83%	1,39%
Loss of interest	51,39%	37,84%	45,83%	2,78%
Postural dizziness	50,00%	41,67%	47,22%	2,78%
Involuntary movements	48,61%	40,00%	51,39%	0,00%
Urinary incontinence	48,61%	20,00%	51,39%	0,00%
Constipation	43,06%	32,26%	55,56%	1,39%
Hearing problems	43,06%	32,26%	54,17%	2,78%
Solitude	40,28%	34,48%	58,33%	1,39%
Difficulty swallowing	38,89%	60,71%	59,72%	1,39%
Aggressiveness	34,72%	52,00%	65,28%	0,00%
Problems/arguments	33,33%	45,83%	65,28%	1,39%
Weight loss for no apparent reason	33,33%	41,67%	66,67%	0,00%
Impulsive behaviours	33,33%	33,33%	65,28%	1,39%
Suicidal thoughts	6,94%	80,00%	90,28%	2,78%

*worried about it: percentages are calculated from the total YES answers

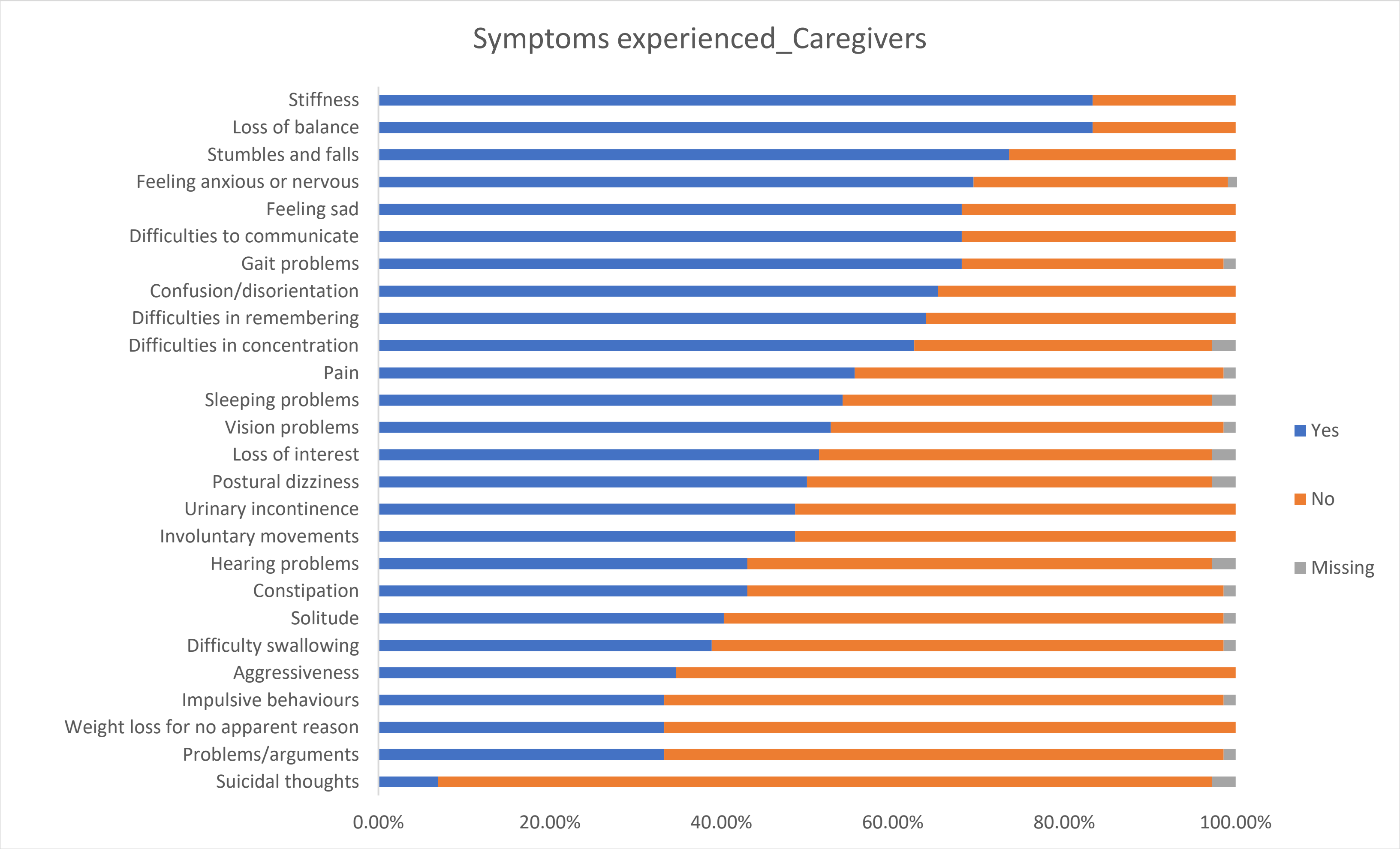

Symptoms experienced_Caregivers
Stiffness
Loss of balance
Stumbles and falls
Feeling anxious or nervous
Feeling sad
Difficulties to communicate
Gait problems
Confusion/disorientation
Difficulties in remembering
Difficulties in concentration
Pain
Sleeping problems
Vision problems
Loss of interest
Postural dizziness
Urinary incontinence
Involuntary movements
Hearing problems
Constipation
Solitude
Difficulty swallowing
Aggressiveness
Impulsive behaviours
Weight loss for no apparent reason
Problems/arguments
Suicidal thoughts
0.00%
20.00%
40.00%
60.00%
80.00%
100.00%
Yes
No
Missing

Most frequent symptoms as reported by the patients

Most frequent (more than ½ of the patients)			
**Motor**	**Non-motor**	**Most worried about**	**Most frequent and worried about**
Stiffness (77,6%)<br>Loss of balance (65,9%)<br>Gait problems (65,9%)<br>Pain (60,0%)<br>Involuntary movements (55,3%)	Feeling sad (71,8%)<br>Feeling anxious or nervous (65,9%)<br>Difficulties in concentration (57,7%)<br>Sleeping problems (56,5%)<br>Difficulties to communicate (55,3%)	Pain (47,1%)<br>Loss of balance (46,4%)<br>Gait problems (42,9%)<br>Stiffness (40,9%)<br>Involuntary movements (40,4%)<br>Feeling anxious or nervous (32,1 %)	Stiffness<br>Loss of balance<br>Gait problems<br>Pain<br>Feeling sad<br>Feeling anxious or nervous

Most frequent symptoms as reported by the caregivers

Most frequent (more than ½ of the caregivers)			
Motor	Non-motor	**Most worried about**	**Most frequent and worried about**
Loss of balance (83,33%)<br>Stiffness (83,33%)<br>Stumbles and falls (73,61%)<br>Gait problems (68,06%)<br>Pain (55,56%)<br>Postural dizziness (50,00%)	Feeling anxious or nervous (69,44%)<br>Difficulties to communicate (68,06%)<br>Feeling sad (68,06%)<br>Confusion/disorientation (65,28%)<br>Difficulties in remembering (63,89%)<br>Difficulties in concentration (62,50%)<br>Sleeping problems (54,17%)<br>Vision problems (52,78%)<br>Loss of interest (51,39%)	Stumbles and falls (56,6%)<br>Loss of balance (51,67%)<br>Stiffness (43,3%)<br>Postural dizziness (41,6%)	- Stiffness<br>- Loss of balance<br>- Stumble and falls<br>- Feeling anxious or nervous<br>- Gait problems<br>- Feeling sad

b. Difficulties in activities of daily living (ADLs)→Corresponding to ST1.3

<u>Question No. 16: (Patients version):</u> Please, could you tell us if you normally find it difficult to perform any of the following daily tasks? Indicate YES or NO?

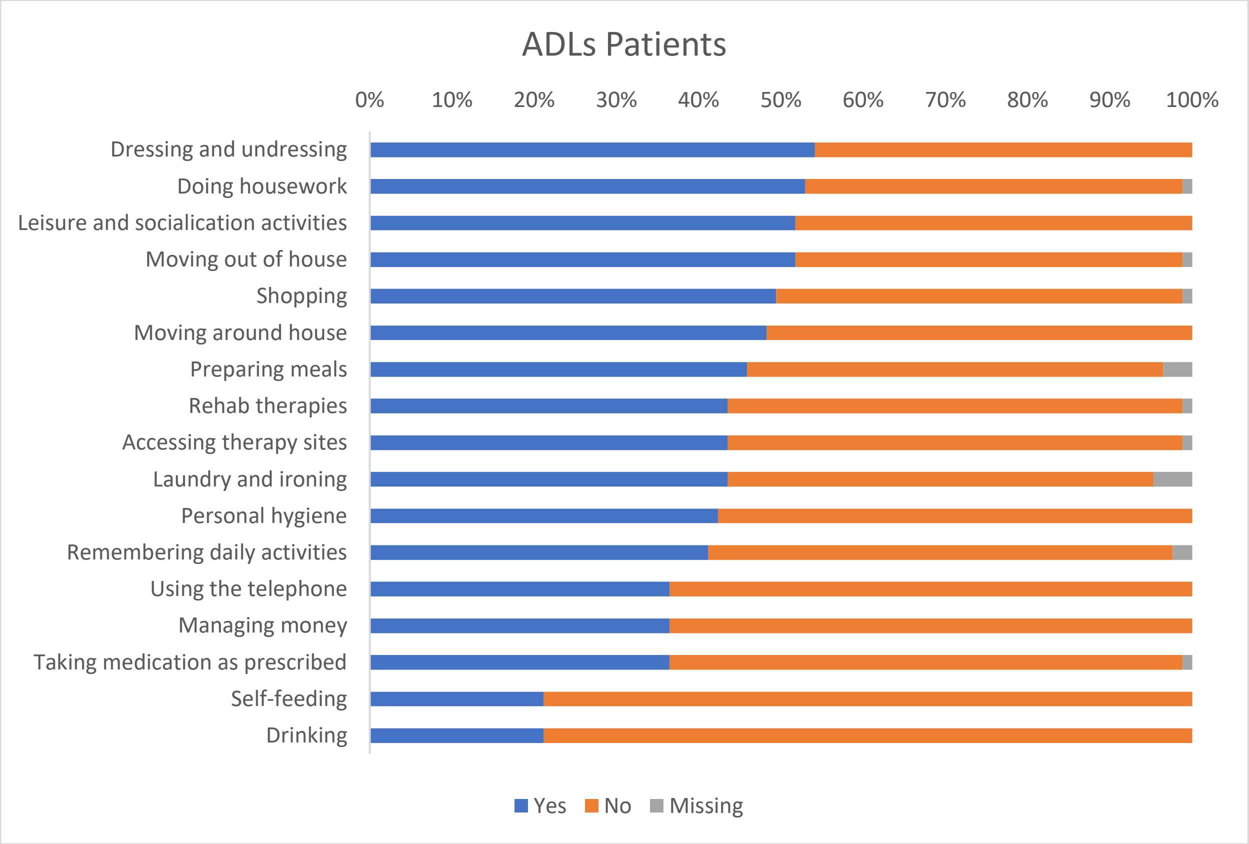

ADL	Patients` answers (n=85)		
	Yes %	No %	Missing %
**Dressing and undressing**	54,12%	45,88%	0,00%
**Doing housework**	52,94%	45,88%	1,18%
**Leisure and socialication activities**	51,76%	48,24%	0,00%
**Moving out of house**	51,76%	47,06%	1,18%
**Shopping**	49,41%	49,41%	1,18%
**Moving around house**	48,24%	51,76%	0,00%
**Preparing meals**	45,88%	50,59%	3,53%
**Rehab therapies**	43,53%	55,29%	1,18%
**Accessing therapy sites**	43,53%	55,29%	1,18%
**Laundry and ironing**	43,53%	51,76%	4,71%
**Personal hygiene**	42,35%	57,65%	0,00%
**Remembering daily activities**	41,18%	56,47%	2,35%
**Using the telephone**	36,47%	63,53%	0,00%
**Managing money**	36,47%	63,53%	0,00%
**Taking medication as prescribed**	36,47%	62,35%	1,18%
**Self-feeding**	21,18%	78,82%	0,00%
**Drinking**	21,18%	78,82%	0,00%

Question No. 19: (caregiver version): Please, could you tell us in which of the following activities of daily life, the person you care for have difficulties. Indicate YES or NO. Please, add on the right side of the table when you have indicated yes, if you have to help the person you care for?

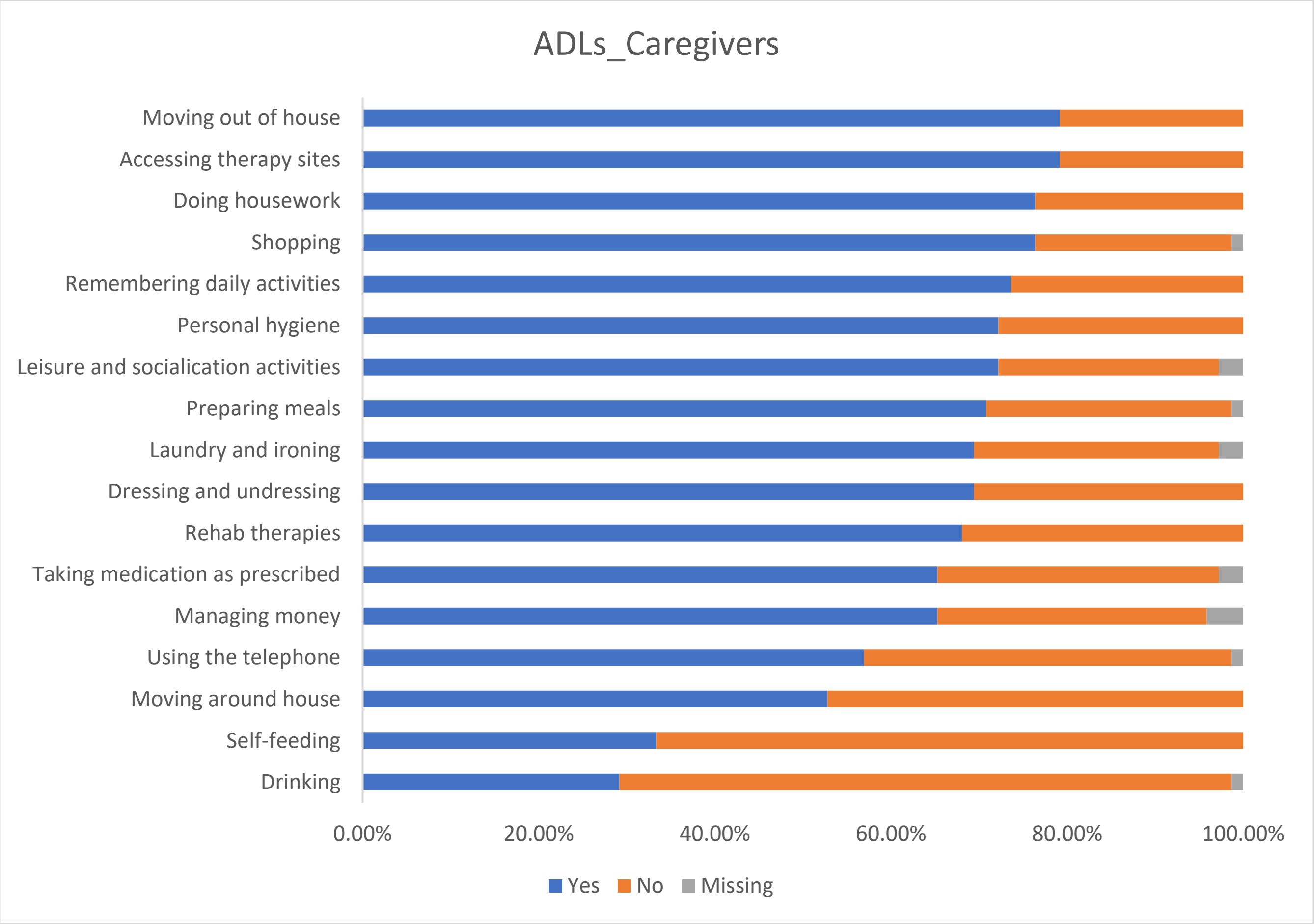

ADL	Caregivers` answers (n=72)			
	Yes%		No %	Missing %
		Needs help*%		
Accessing therapy sites	79,17%	75,44%	20,83%	0,00%
Moving out of house	79,17%	70,18%	20,83%	0,00%
Shopping	76,39%	78,20%	22,22%	1,39%
Doing housework	76,39%	67,27%	23,61%	0,00%
Remembering daily activities	73,61%	75,47%	26,39%	0,00%
Leisure and socialication activities	72,22%	73,08%	25,00%	2,78%
Personal hygiene	72,22%	71,15%	27,78%	0,00%
Preparing meals	70,83%	66,67%	27,78%	1,39%
Dressing and undressing	69,44%	74,00%	30,56%	0,00%
Laundry and ironing	69,44%	68,00%	27,78%	2,78%
Rehab therapies	68,06%	67,35%	31,94%	0,00%
Managing money	65,28%	72,34%	30,56%	4,17%
Taking medication as prescribed	65,28%	68,09%	31,94%	2,78%
Using the telephone	56,94%	68,29%	41,67%	1,39%
Moving around house	52,78%	55,26%	47,22%	0,00%
Self-feeding	33,33%	66,67%	66,67%	0,00%
Drinking	29,17%	57,14%	69,44%	1,39%

*Needs help: Percentages are calculated from the total YES answers

c. **Caregiver family burden→ Corresponding to ST1.3**
Questions 16a-16g (abbreviated 7-items Zarit scale)
16a: Do you think that because of the time you spend with the person you care for you don't have enough time for yourself?
16b: Do you feel burdened by trying to juggle your caring tasks with other responsibilities (work, family)?
16c: Do you feel that caring negatively affects your relationship with other people?
16d: Do you think your health has worsened because of caring for person of interest?
16e: Do you feel tense around the person you care for?
16f: Do you feel like you have lost control of your life since your family member's illness began?
16g: Overall, how much of a burden do you experience in caring for that person?

Caregiver Family Burden (abbreviated Zarit scale)

			Percent	Valid Percent	Cumulative Percent
Valid	Burden	41	56,9	57,7	57,7
	No burden	30	41,7	42,3	100,0
	Total	71	98,6	100,0	
Missing	System	1	1,4		
Total		72	100,0		

SPSS Statistics 27

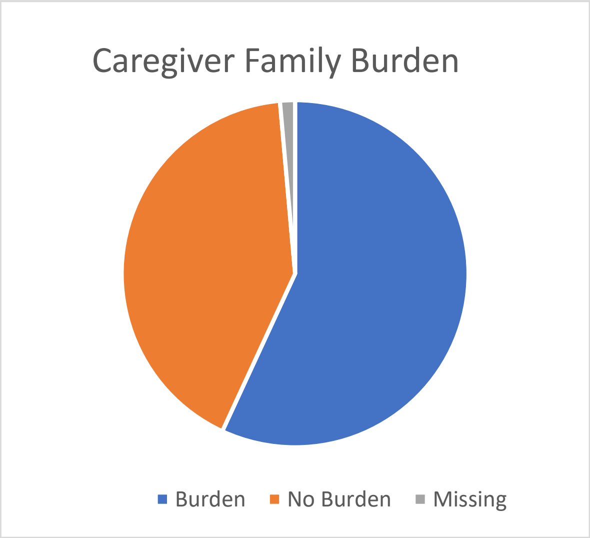

Supplement: Multimedia Appendix 1 [file formative_v6i11e39199_app1.pdf]
