# Supplementary material for: End Users’ and Other Stakeholders’ Needs and Requirements in the Development of a Personalized Integrated Care Platform (PROCare4Life) for Older People With Dementia or Parkinson Disease: Mixed Methods Study
Source: JMIR Form Res. 2022 Nov 30;6(11):e39199. doi: 10.2196/39199 (PMC9752454; doi:10.2196/39199)

(Appendix 2)

Results from the web-based surveys corresponding to theme 2 “Thoughts about the Platform Technology”

a. Device acceptance → Corresponding to ST2.2

<u>Question No. 31</u> (patients` version): So that your social-health professionals could provide you with better care and treatments, which of the following devices and systems would you accept to use?

DEVICE ACCEPTANCE - PATIENTS (n=85)

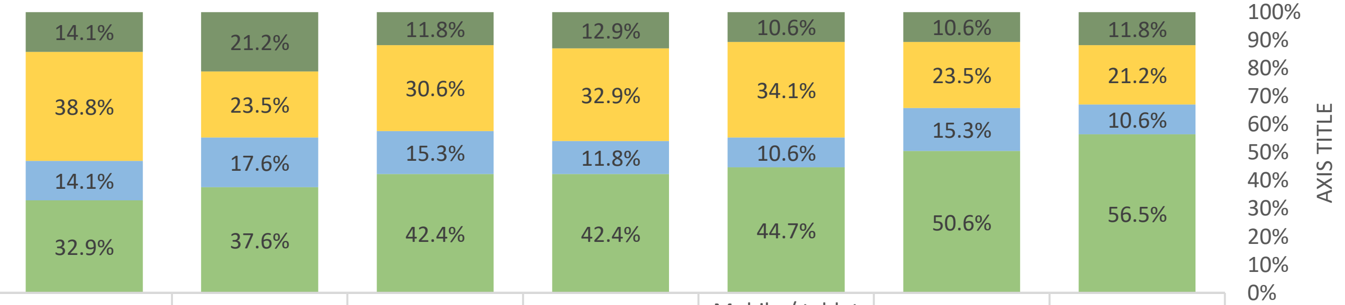

	Cameras	Object sensors	Website	Smart TV	Mobile / tablet app	Stationary device	Wearable device
Missing / No opinion	14.1%	21.2%	11.8%	12.9%	10.6%	10.6%	11.8%
I would not use it / I would prefer not to use it	38.8%	23.5%	30.6%	32.9%	34.1%	23.5%	21.2%
I am not sure if I will use it	14.1%	17.6%	15.3%	11.8%	10.6%	15.3%	10.6%
I would like / love to use it	32.9%	37.6%	42.4%	42.4%	44.7%	50.6%	56.5%

<u>Question No. 30</u> (caregivers` version): So that your social-health-care professionals could provide the person you care for, with a better care and treatments, which of the following devices and systems would you accept to?

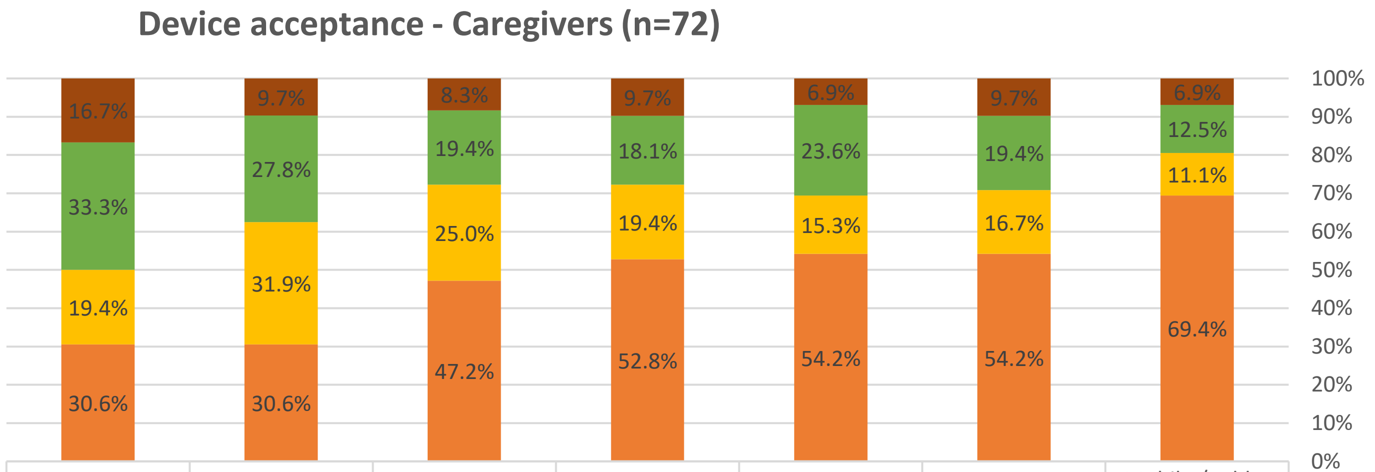

	Object sensors	Cameras	Smart TV	Stationary device	Wearable device	Website	Mobile / tablet app
Missing / No opinion	16.7%	9.7%	8.3%	9.7%	6.9%	9.7%	6.9%
I would not use it / I would prefer not to use it	33.3%	27.8%	19.4%	18.1%	23.6%	19.4%	12.5%
I am not sure if I will use it	19.4%	31.9%	25.0%	19.4%	15.3%	16.7%	11.1%
I would like / love to use it	30.6%	30.6%	47.2%	52.8%	54.2%	54.2%	69.4%

b. Expected Benefits → Corresponding to ST2.3

<u>Questions No. 66 till 71</u> (patients` version)

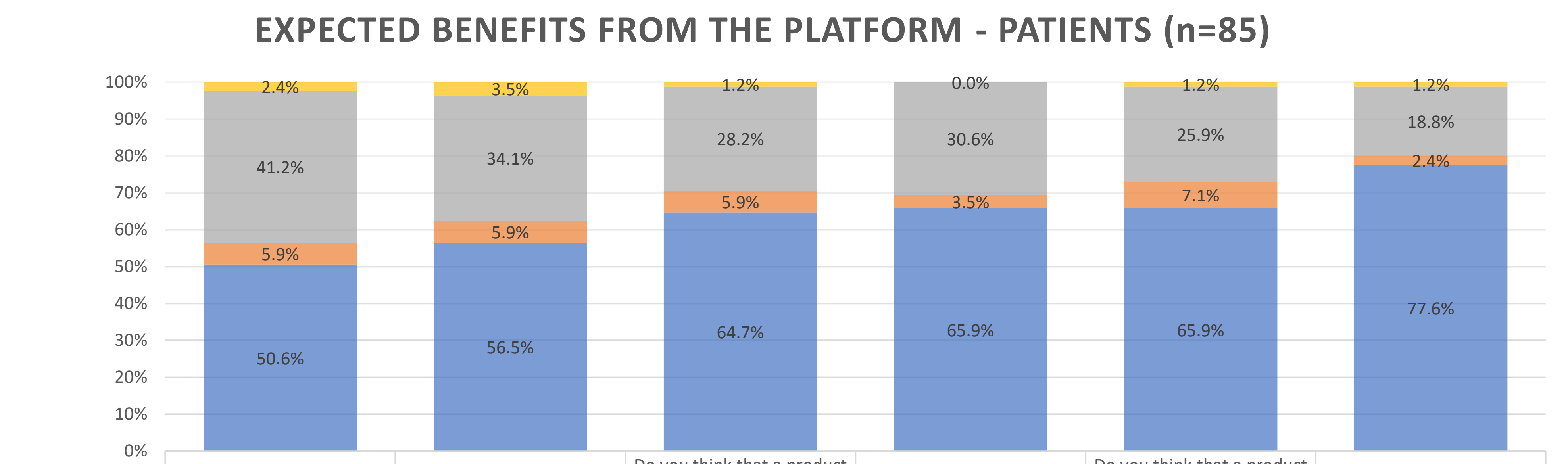

	Do you think that a product such as PROCare4Life could be a valid tool to respond your needs?	Do you think that a product such as PROCare4Life might reduce the severity of some symptoms (e.g. anxiety, depression, falls)?	Do you think that a product such as PROCare4Life, which includes a communication service, might contribute to improve patient's social participation?	Do you think that the system might contribute to increase the patient's perception of empowerment?	Do you think that a product such as PROCare4Life, with personalised recommendations, might contribute to improve a patients' mental or physical condition?	Do you think that a product such as PROCare4Life might contribute to increasing feelings of safety / autonomy of patients in their homes?
Missing	2.4%	3.5%	1.2%	0.0%	1.2%	1.2%
I do not know	41.2%	34.1%	28.2%	30.6%	25.9%	18.8%
No	5.9%	5.9%	5.9%	3.5%	7.1%	2.4%
Yes	50.6%	56.5%	64.7%	65.9%	65.9%	77.6%

<u>Questions No. 68 till 73</u> (caregivers` version)

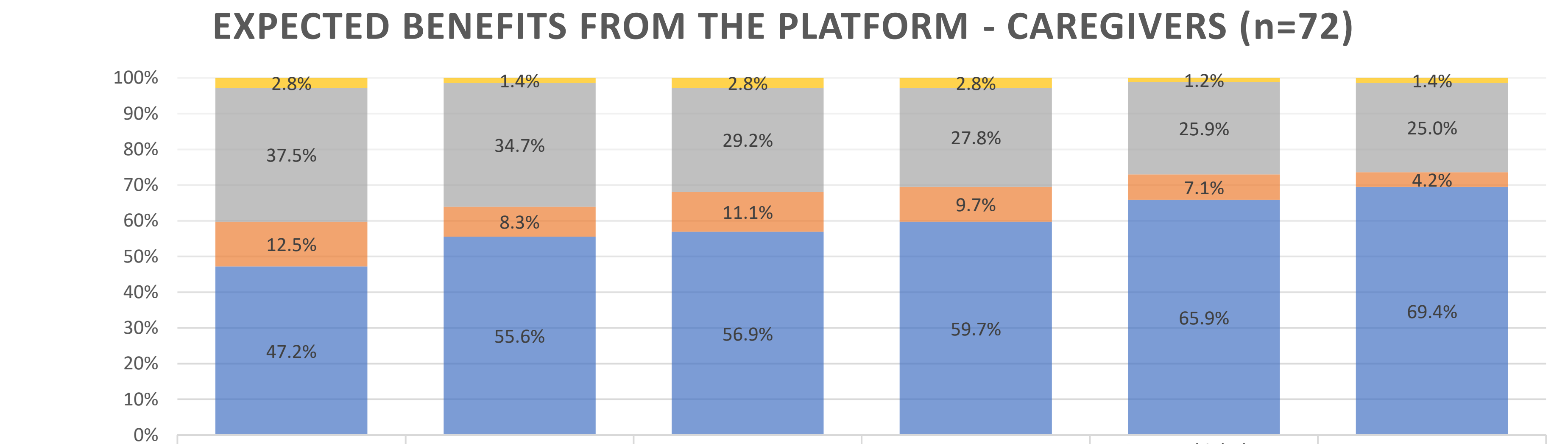

	Do you think that a product such as PROCare4Life might reduce the severity of some symptoms (e.g. anxiety, depression, falls)?	Do you think that a product such as PROCare4Life could be a valid tool to respond your needs?	Do you think that a product such as PROCare4Life, which includes a communication service, might contribute to improve patient's social participation?	Do you think that the system might contribute to increase the patient's perception of empowerment?	Do you think that a product such as PROCare4Life, with personalised recommendations, might contribute to improve a patients' mental or physical condition?	Do you think that a product such as PROCare4Life might contribute to increasing feelings of safety / autonomy of patients in their homes?
Missing	2.8%	1.4%	2.8%	2.8%	1.2%	1.4%
I do not know	37.5%	34.7%	29.2%	27.8%	25.9%	25.0%
No	12.5%	8.3%	11.1%	9.7%	7.1%	4.2%
Yes	47.2%	55.6%	56.9%	59.7%	65.9%	69.4%

c. **Willingness to Invest →Corresponding to ST2.5**

<u>Question No. 72</u> (patient version) and **<u>No. 74</u>** (caregiver version): Would you pay to have a system like this one?

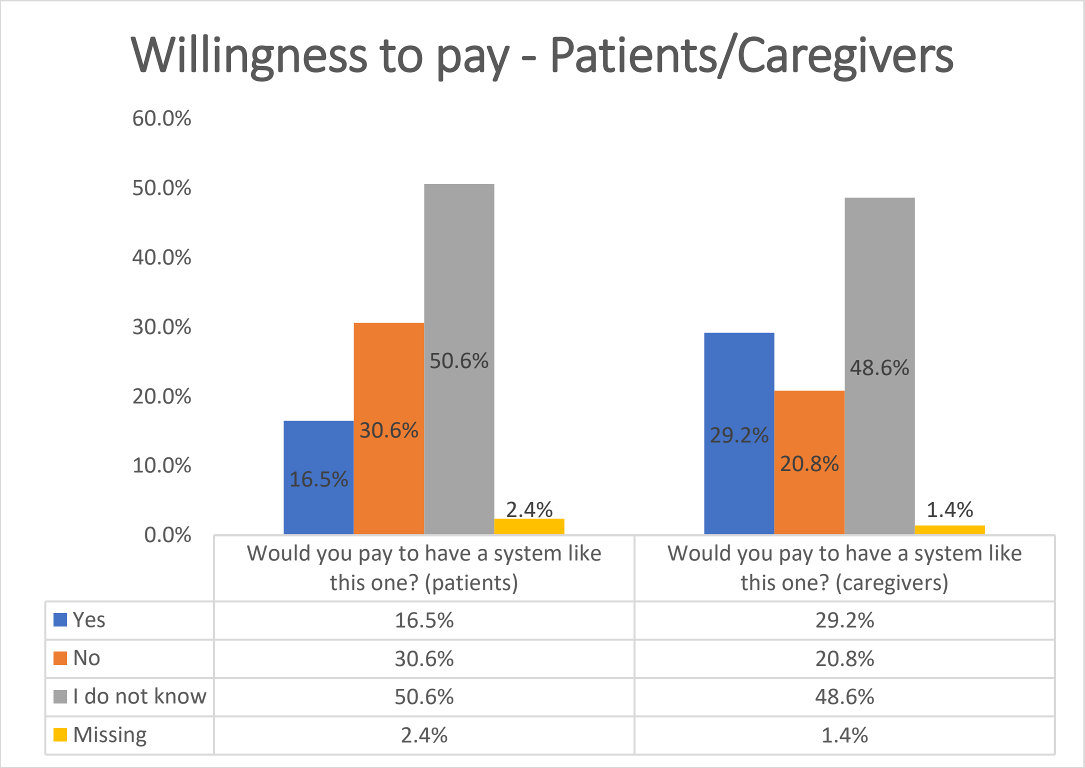

Supplement: Multimedia Appendix 2 [file formative_v6i11e39199_app2.pdf]
