# Supplementary material for: End Users’ and Other Stakeholders’ Needs and Requirements in the Development of a Personalized Integrated Care Platform (PROCare4Life) for Older People With Dementia or Parkinson Disease: Mixed Methods Study
Source: JMIR Form Res. 2022 Nov 30;6(11):e39199. doi: 10.2196/39199 (PMC9752454; doi:10.2196/39199)
Supplement: Multimedia Appendix 3 [file formative_v6i11e39199_app3.pdf]

(Appendix 3)

Results from the web-based surveys corresponding to theme 3 “Desired Properties”

a. **Preferred platform functionalities → Corresponding to ST3.1, ST3.2, ST3,3, ST3.4, and ST3.5**

The following tables and figures represent answers from both patients and caregivers regarding preferences of different platform functionalities

Questions No.39-49 (Patients version): Which of the following platform functionalities, would you perceive as helpful for you?

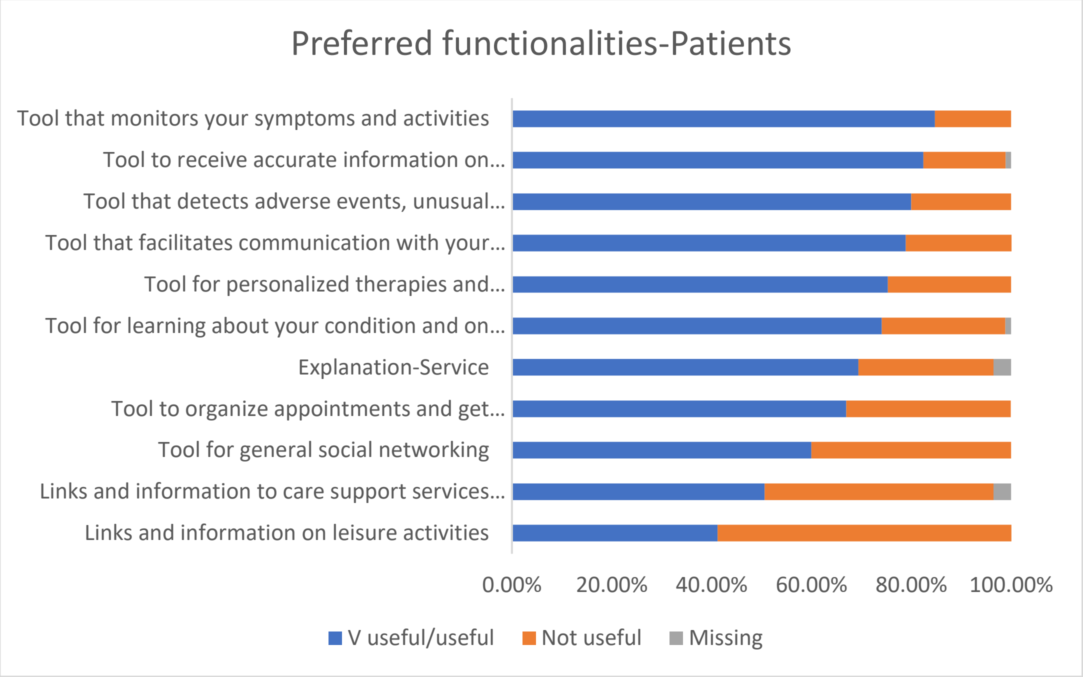

Functionality	Patients` answers (n=85)		
	Very useful/useful %	Not useful %	Missing %
**Links and information on leisure activities**	41,20%	58,8%	0,0%
**Links and information to care support services in your area**	50,60%	45,9%	3,5%
**Tool for general social networking**	60%	40,0%	0,0%
**Tool to organize appointments and get reminders that make aware of activities you need to do**	67%	32,9%	0,0%
**Explanation-Service**	69,40%	27,1%	3,5%
**Tool for learning about your condition and on how to deal with symptoms**	74,10%	24,7%	1,2%
**Tool for personalized therapies and recommendations adapted to your preferences and your condition evolution**	75,30%	24,7%	0,0%
**Tool that facilitates communication with your social-health professionals at a distance**	78,90%	21,2%	0,0%
**Tool that detects adverse events, unusual activities or movements**	80%	20,0%	0,0%
**Tool to receive accurate information on physiological status**	82,40%	16,5%	1,1%
**Tool that monitors your symptoms and activities**	84,70%	15,3%	0,0%

<u>Questions No. 37-49</u> (Caregivers version): Which of the following platform functionalities, would you perceive as helpful for you as a caregiver?

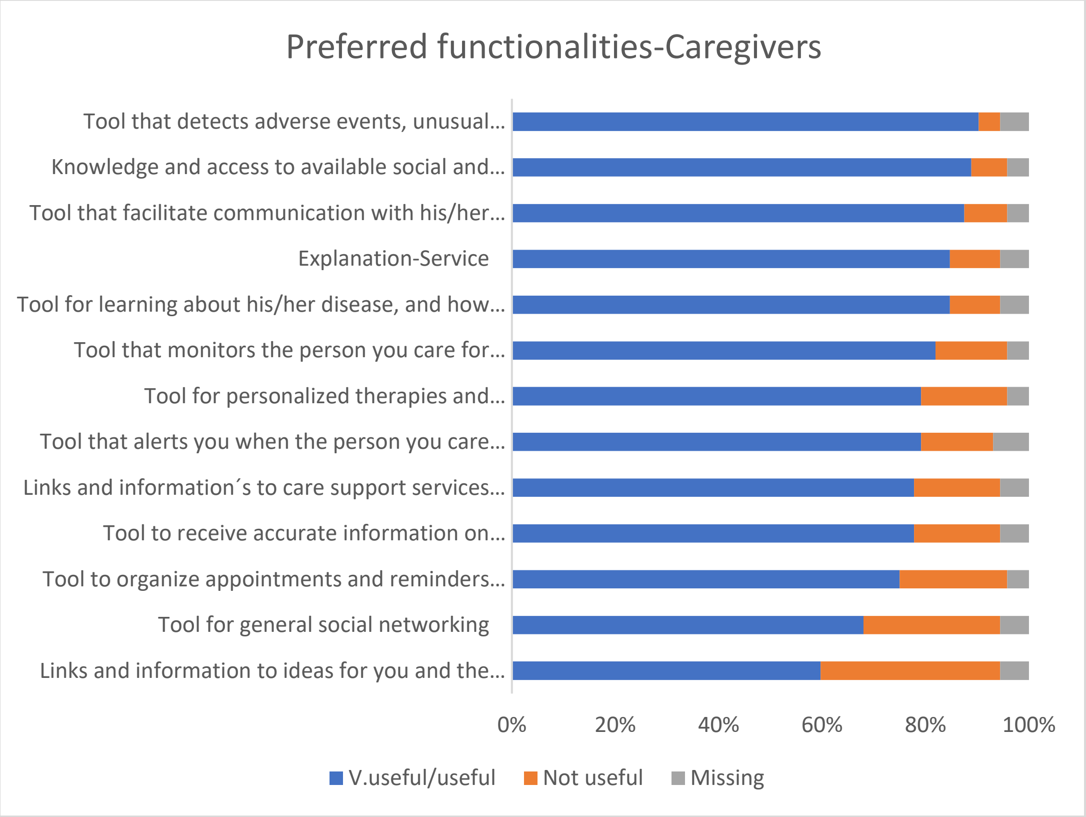

**Functionality**	**Caregivers` answers (n=72)**		
	**V.useful/useful %**	**Not useful %**	**Missing %**
**Links and information to ideas for you and the person you care for leisure time**	59,70%	34,7%	5,6%
**Tool for general social networking**	68%	26,4%	5,6%
**Tool to organize appointments and reminders together with the person you care for**	75%	20,8%	4,2%
**Tool to receive accurate information on physiological status**	77,80%	16,7%	5,6%
**Links and information´s to care support services in your area**	77,80%	16,7%	5,6%
**Tool that alerts you when the person you care for is not doing an important activity**	79,20%	13,9%	6,9%
**Tool for personalized therapies and recommendations, adapted to his/her preferences and condition evolution**	79,20%	16,7%	4,2%
**Tool that monitors the person you care for symptoms and activities and provides information to his/her social-health professionals, so that they can adjust treatments better**	81,90%	13,9%	4,2%
**Tool for learning about his/her disease, and how to deal with his/her symptoms, or how to look after yourself as a caregiver**	84,70%	9,7%	5,6%
**Explanation-Service**	84,80%	9,7%	5,6%
**Tool that facilitate communication with his/her social-health professionals from a distance**	87,50%	8,3%	4,2%
**Knowledge and access to available social and healthcare resources**	88,90%	6,9%	4,2%
**Tool that detects adverse events, unusual activities or movements**	90,30%	4,2%	5,6%

b. Preferred platform features→ Corresponding to ST3.6, ST3.7, and ST3.8

<u>Questions No. 51-61</u> (patient version): In order to design a platform that users like, we would like to know which of the following features are perceived as important to you?

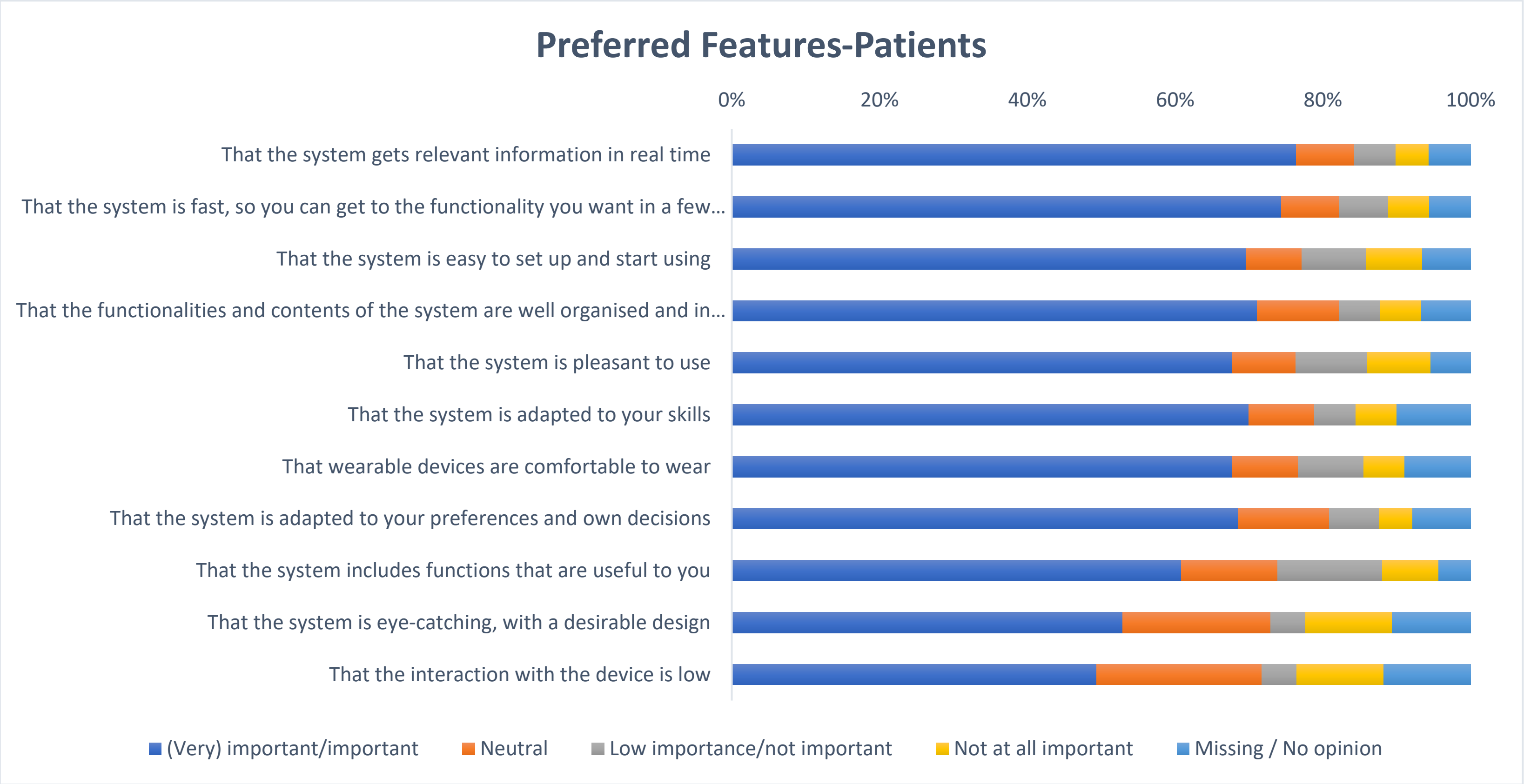

Patients:

**Feature**	**Patients` answers (n=85)**				
	**Very important/ important %**	**Neutral %**	**Low importance %**	**Not at all important %**	**Missing / No opinion %**
That the system gets relevant information in real time	80,0%	8,2%	5,9%	4,7%	5,9%
That the system is fast, so you can get to the functionality you want in a few steps	78,8%	8,2%	7,1%	5,9%	5,9%
That the system is easy to set up and start using	75,3%	8,2%	9,4%	8,2%	7,1%
That the functionalities and contents of the system are well organised and in consequence easy to find	75,3%	11,8%	5,9%	5,9%	7,1%
That the system is pleasant to use	74,1%	9,4%	10,6%	9,4%	5,9%
That the system is adapted to your skills	74,1%	9,4%	5,9%	5,9%	10,6%
That wearable devices are comfortable to wear	71,8%	9,4%	9,4%	5,9%	9,4%
That the system is adapted to your preferences and own decisions	71,8%	12,9%	7,1%	4,7%	8,2%
That the system includes functions that are useful to you	65,9%	14,1%	15,3%	8,2%	4,7%
That the system is eye-catching, with a desirable design	52,9%	20,0%	4,7%	11,8%	10,6%
That the interaction with the device is low	49,4%	22,4%	4,7%	11,8%	11,8%

Questions No. 51-62 (Caregivers version): In order to design a system that users like, which of the following features are perceived as important to you?

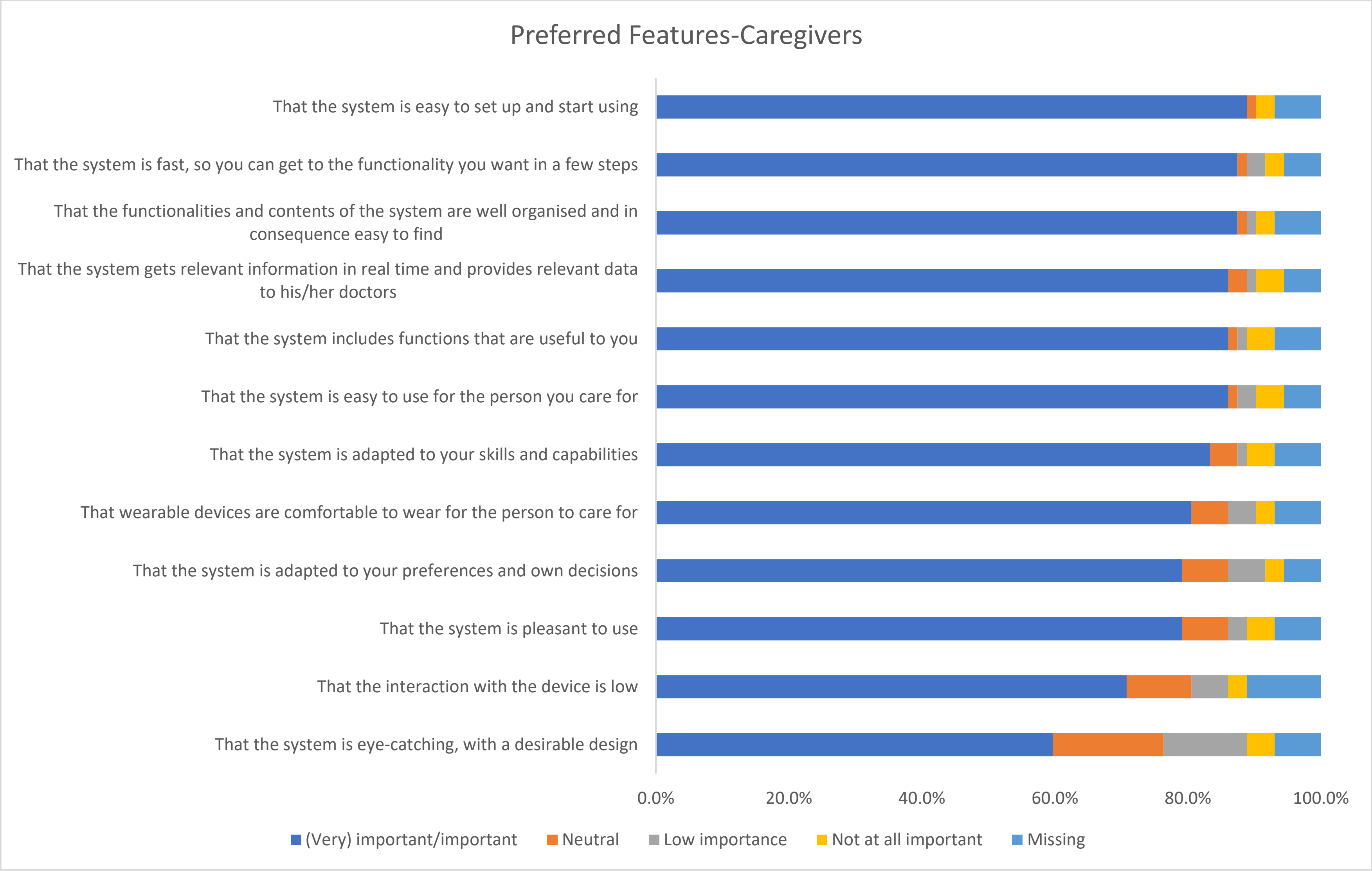

Caregivers:

Feature	Caregivers` answers (n=72)				
	**Very important/ important %**	**Neutral %**	**Low importance %**	**Not at all important %**	**Missing / No opinion %**
That the system is easy to set up and start using	88,9%	1,4%	0,0%	2,8%	6,9%
That the system is fast, so you can get to the functionality you want in a few steps	87,5%	1,4%	2,8%	2,8%	5,6%
That the functionalities and contents of the system are well organized and in consequence easy to find	87,5%	1,4%	1,4%	2,8%	6,9%
That the system gets relevant information in real time, and provides relevant information to her/his doctor	86,1%	2,8%	1,4%	4,2%	5,6%
That the system includes functions that are useful to you	86,1%	1,4%	1,4%	4,2%	6,9%
That the system is easy to use for the person you care for	86,1%	1,4%	2,8%	4,2%	5,6%
That the system is adapted to your skills and capabilities	83,3%	4,2%	1,4%	4,2%	6,9%
That wearable devices are comfortable to wear for the person you care for	80,6%	5,6%	4,2%	2,8%	6,9%
That the system is adapted to your preferences and own decisions	79,2%	6,9%	5,6%	2,8%	5,6%
That the system is pleasant to use	79,2%	6,9%	2,8%	4,2%	6,9%
That the interaction with the device is low	70,8%	9,7%	5,6%	2,8%	11,1%
That the system is eye-catching, with a desirable design	59,7%	16,7%	12,5%	4,2%	6,9%
